# A global analysis of alternative tillage and crop establishment practices for economically and environmentally efficient rice production

**DOI:** 10.1038/s41598-017-09742-9

**Published:** 2017-08-24

**Authors:** Debashis Chakraborty, Jagdish Kumar Ladha, Dharamvir Singh Rana, Mangi Lal Jat, Mahesh Kumar Gathala, Sudhir Yadav, Adusumilli Narayana Rao, Mugadoli S. Ramesha, Anitha Raman

**Affiliations:** 1 ICAR-Indian Agricultural Research Institute, New Delhi, 110012 India; 2 International Rice Research Institute, Las Banos, Philippines; 3 International Rice Research Institute, New Delhi, 110012 India; 4 International Maize and Wheat Improvement Center, New Delhi, 110012 India; 5 International Maize and Wheat Improvement Center-Bangladesh, Dhaka, 1212 Bangladesh; 6 International Rice Research Institute, ICRISAT, Patancheru, 500033 India

## Abstract

Alternative tillage and rice establishment options should aim at less water and labor to produce similar or improved yields compared with traditional puddled-transplanted rice cultivation. The relative performance of these practices in terms of yield, water input, and economics varies across rice-growing regions. A global meta and mixed model analysis was performed, using a dataset involving 323 on-station and 9 on-farm studies (a total of 3878 paired data), to evaluate the yield, water input, greenhouse gas emissions, and cost and net return with five major tillage/crop establishment options. Shifting from transplanting to direct-seeding was advantageous but the change from conventional to zero or reduced tillage reduced yields. Direct-seeded rice under wet tillage was the best alternative with yield advantages of 1.3–4.7% (p < 0.05) and higher net economic return of 13% (p < 0.05), accompanied by savings of water by 15% (p < 0.05) and a reduction in cost by 2.4–8.8%. Direct-seeding under zero tillage was another potential alternative with high savings in water input and cost of cultivation, with no yield penalty. The alternative practices reduced methane emissions but increased nitrous oxide emissions. Soil texture plays a key role in relative yield advantages, and therefore refinement of the practice to suit a specific agro-ecosystem is needed.

## Introduction

Rice is one of the most important food crops in the world. It is a staple for more than half of the global population, with 11% of cultivated land^1^. Worldwide, rice is grown on 159 million hectares (Mha), with an annual production of 703.8 million tons (Mt) of paddy^2^. About 90% of the world’s rice is grown and produced in Asia, which is projected at 676.5 Mt in 2016 from 143 Mha of area^3^. Global per capita rice consumption is hovering around 57 kg yr^−1^ ^4^, and therefore an increase in total consumption follows the rate of growth of population. The global population is expected to be 9.7 billion by 2050, with projected need for a 60% increase in agricultural production to meet the rising population and changing diets^5^. The additional production must also consider the impact of climate change, which is estimated to reduce rice production in 2050 by 11.9% to 13.5% of the production in 2000^6^. The possibility of expanding the area under rice in the near future is limited^7^. Therefore, this additional rice production needed has to come mainly from a productivity gain.

About 77% of global rice is grown in wetlands with intensive tillage (puddling or wet tillage) followed by transplanting^8, 9^. This practice of conventional tillage and crop establishment is labor-, water-, capital-, and energy-intensive, and it is becoming less profitable as these resources are becoming increasingly scarce^10^. Puddling has an adverse impact on the succeeding crop, including deterioration of soil structure^11^, poor crop establishment^12, 13^, and limited root growth due to subsurface compaction^14^. Water use in agriculture is projected to increase by 20% in 2050, and irrigation accounts for 70% of global water withdrawals^15^, whereas water availability for agriculture is projected to decrease by 10% by 2025^16^. Rice alone consumes about 50% of total irrigation water in Asia, and accounts for 24–30% of the withdrawal of world total freshwater^17, 18^. Puddling accounts for 30% of the rice water requirement, with a major proportion being lost through percolation and evaporation^19^. It is estimated that, by 2025, 17 and 22 Mha of irrigated rice area in Asia will have physical and economic water scarcity^20^, necessitating water-saving options to be practiced widely. Rice transplanting, a job done manually, is highly labor-intensive, requiring 25–50 person-days ha^−1^. In Asia, it is largely done by women and the drudgery leads to many occupational hazards for them^21^. Rapid economic growth in Asia has increased the demand for labor in non-agricultural sectors, resulting in reduced labor availability for agriculture, especially during the peak season^22^, and increased labor wages^23^. The size of the workforce in agriculture declined by nearly 30 million between 2004–05 and 2011–12, and a considerable decline in labor availability has been reported in paddy-growing areas in India^22^. A seasonally flooded paddy field is a major source of methane (10–13% of anthropogenic methane in the atmosphere) and contributes significantly to global GHG emissions from agriculture^24^.

These factors demand a major shift from puddled transplanting to alternative methods of establishing rice. Consequently, several options of mechanical direct-seeding and transplanting under unpuddled/non-flooded conditions have been developed and evaluated by researchers in collaborations with farmers^10, 25, 26^. Notably, direct-seeded rice with or without tillage has emerged as a viable alternative to drudgery, particularly of women, and to energy-intensive puddling/transplanting. Hundreds of individual on-station and on-farm trials have compared yields and other performance parameters of various tillage/rice establishment options, but an attempt to synthesize information on a global scale is critically lacking. Such a synthesis is essential to (a) prioritize research and development issues, including precise technology targeting, and (b) articulate policy and institutional measures to facilitate large-scale adoption.

We performed a comprehensive meta-analysis and mixed model analysis on data from on-station (1980–2016) and on-farm studies (2009–2016) carried out globally to compare the performances of five major options of rice tillage and crop establishment (CE) options with that of conventional tillage (puddling) and transplanting. Performance parameters considered in the meta-analysis and the mixed model analysis included (a) grain yield, (b) water input, (c) GHG emissions, and (d) cost of cultivation and net economic returns.

## Results

### Comparisons of grain yield among various tillage and CE options (a) on-station vs on-farm and (b) dry season vs wet season

**Figure 1:**
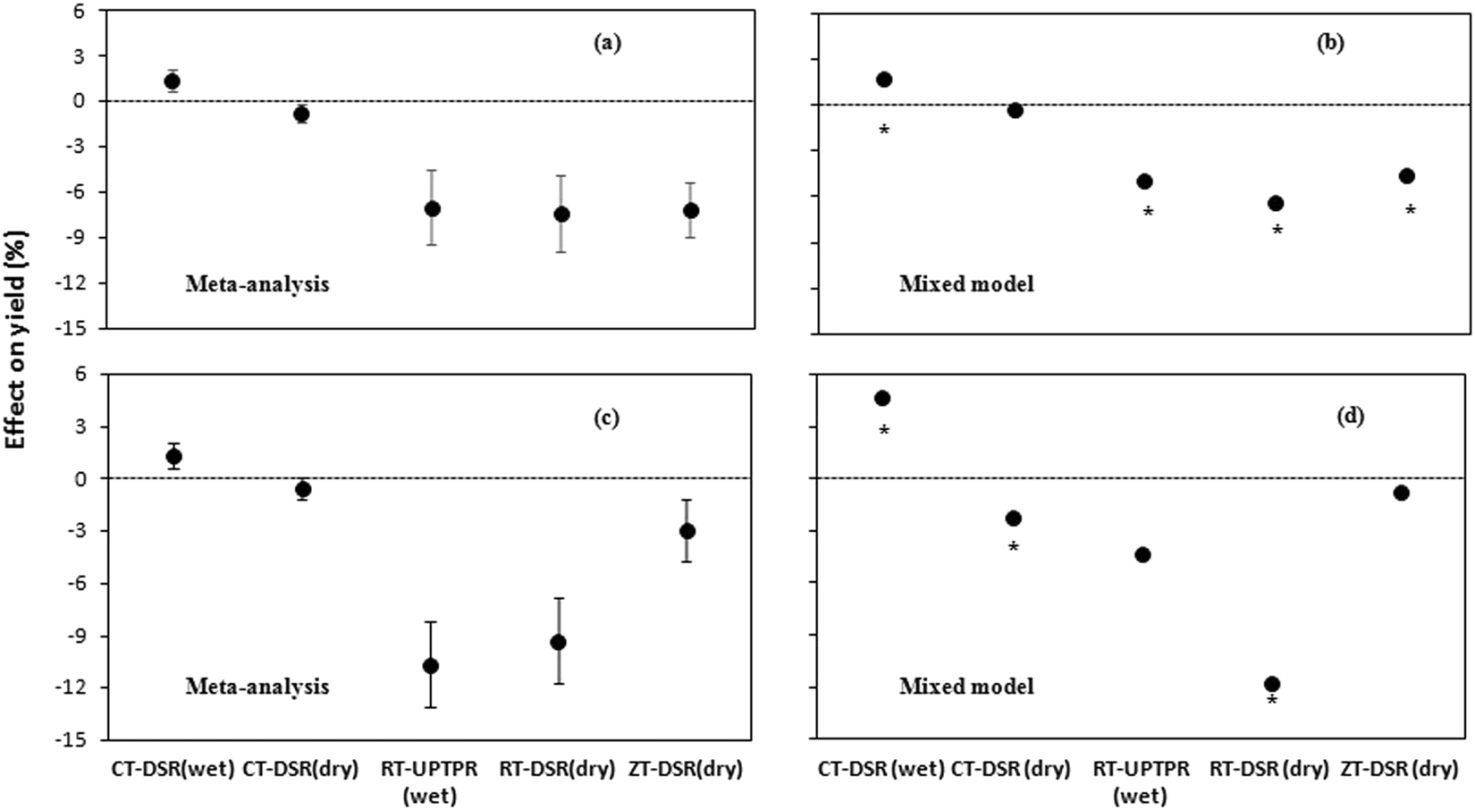
Rice grain yield comparison in five tillage/CE options with CT-TPR(wet). Meta-analysis and mixed model using (**a**,**b**) whole dataset (on-station plus on-farm) and (**c**,**d**) on-station dataset only. Error bars in meta-analysis indicate 95% confidence intervals, where difference in yield is considered significant if the 95% CI does not cover zero. In mixed model, * indicates significant yield difference at p < 0.05.

The meta- and mixed model analysis of the whole dataset (on-station plus on-farm) showed either an increase or no change in grain yield when only the crop establishment by transplanting (TPR) under conventional tillage (CT) was substituted by direct-seeded rice (DSR) either in wet (CT-DSR wet) or dry (CT-DSR dry) conditions (Fig. 1a,b). Compared with the CT-TPR(wet), the CT-DSR(wet) had a yield advantage of 1.4% in meta-analysis and 1.8% in mixed model analysis. Yield in CT-DSR(dry) was lower by 0.7% in meta-analysis but remained unchanged when using the mixed model. On the other hand, rice yield declined when both CT and TPR were substituted by reduced tillage (RT) or zero tillage (ZT) and DSR (Fig. 1a,b). The yields of RT-UPTPR(wet), RT-DSR(dry), and ZT-DSR(dry) did not differ from each other but, compared with the yield of CT-TPR(wet), three options incurred yield losses ranging from 7.0% to 7.4% according to the meta-analysis, and 4.6–6.3% of CT-TPR(wet) by mixed model analysis of both on-station and on-farm data. The on-station data suggest a similar trend (Fig. 1c,d). Clearly, CT-DSR(wet) had yield gains of 1.4% in meta-analysis or 4.7% in the mixed model compared with CT-TPR(wet). Yield in CT-DSR(dry) remained unchanged in the meta-analysis but was lower by 2.3% as estimated by the mixed model. Compared with CT-TPR(wet), RT-UPTPR(wet) and ZT-DSR(dry) had no yield differences in the mixed model but differed significantly in meta-analysis. Lower yield in RT-DSR(dry) was indicated by both analyses. In meta-analysis, ZT-DSR(dry) showed higher yield than RT-UPTPR(wet) and RT-DSR(dry) and it was also higher than RT-DSR(dry) in the mixed model.

The seasonal comparison of different tillage/CE options showed that the overall yield performance was superior in the dry season (increases of 2.8%) compared with the wet season (decreases of 0.8%) (Table 1). The yield of CT-DSR(wet), which was not different from that of CT-TPR(wet) in the wet season, increased by 4.4% when dry season data were analyzed. Likewise, the yield of RT/ZT-DSR(dry) in the dry season was 5.3% higher compared with a decrease of 4.5% in the wet season. However, the yield of RT-UPTPR(wet), which had losses in both seasons, showed a contrasting trend, with higher (−19.1%) yield loss in the dry season than in the wet season (−9.7%) (Table 1).

**Table 1:** Seasonal influence on rice yield differences under alternative tillage and crop establishment practices.

Tillage and CE options	Season	
	Dry	Wet
Overall	2.8 (1.7 to 3.9)*	−0.8 (−1.3 to 0.2)*
CT-DSR(wet)	4.4 (3.3 to 5.5)*	0.1 (0.6 to 0.9)
CT-DSR(dry)	−0.7 (−3.1 to 1.8)	0.2 (−0.6 to 0.9)
RT-UPTPR(wet)	−19.1 (−26.7 to −10.7)*	−9.7 (−12.2 to −7.2)*
RT/ZT-DSR(dry)	5.3 (1.0 to 9.7)*	−4.5 (−6.1 to −2.8)*

Mean values are given with 95% confidence intervals in parentheses. * indicates significant at p < 0.05.

### Comparisons of grain yield among various tillage/CE options in response to soil texture: (a) conventional tillage vs zero tillage, (b) transplanting vs direct-seeding

Soil texture-wise analysis showed a mixed trend. All the tillage/CE options had yield response ranging from negative to positive (−14.7% to + 14.2%) in varying soil texture-groups except RT-UPTPR(wet), which had negative yield responses (−7.3% to −15.3%) in all the soil texture-groups (Table 2). Clayey to loamy groups either had non-significant effects (−0.9% in clayey to −0.5% in medium loamy) to significantly positive effects (3.1% to 3.4% in moderately fine or moderately coarse loamy soils) in CT-DSR(wet), but sandy soil groups showed a 14.7% lower yield. CT-DSR(dry) had negative yield of 3.2% in clayey soils and a positive yield of 0.9% in loamy groups of soils. RT-DSR(dry) yields varied from significantly positive of 14.2% in clayey groups to significantly negative of 8.5% to 13.2% in finer to coarser loamy texture-groups. The yield of ZT-DSR(dry) remained unchanged in all except that a significantly negative change of 10.2% was obtained in loamy moderate-texture soil (Table 2).

**Table 2:** Change (% of CT-TPR) in rice grain yield in five tillage and crop establishment options as influenced by soil texture-groups.

Tillage and CE options	Texture groups				
	Clayey (fine)	Loamy (moderately fine)	Loamy (medium)	Loamy (moderately coarse)	Sandy (coarse)
CT-DSR(wet)	−0.9 (−2.1 to −0.4)	3.1 (2.2 to 4.0)*	−0.5 (−2.7 to 1.8)	3.4 (1.8 to 4.9)*	−14.7 (−17.9 to −11.3)*
CT-DSR(dry)	−3.2 (−4.9 to −1.4)*	0.9 (0.04 to 1.8)*	−2.6 (−4.3 to −0.9)*	−0.8 (−2.2 to −0.5)	−1.7 (−4.0 to −0.7)
RT-UPTPR(wet)	−15.3 (−20.4 to −9.8)*	−7.3 (−11.6 to−2.9)*	−10.9 (−17.4 to −3.8)*	−11.1 (−14.9 to−7.2)*	—
RT-DSR(dry)	14.2 (2.0 to 27.7)*	−8.5 (−16.6 to 0.3)	−10.2 (−16.6 to −3.3)*	−13.2 (−17.3 to −9.0)*	—
ZT-DSR(dry)	−1.7 (−6.3 to 3.1)	−0.1 (−2.4 to 2.3)	−0.05 (−4.0 to 4.0)	−10.2 (−13.1 to −7.1)*	—

Mean values are given with 95% confidence intervals in parentheses. * indicates significant at p < 0.05.

**Figure 2:**
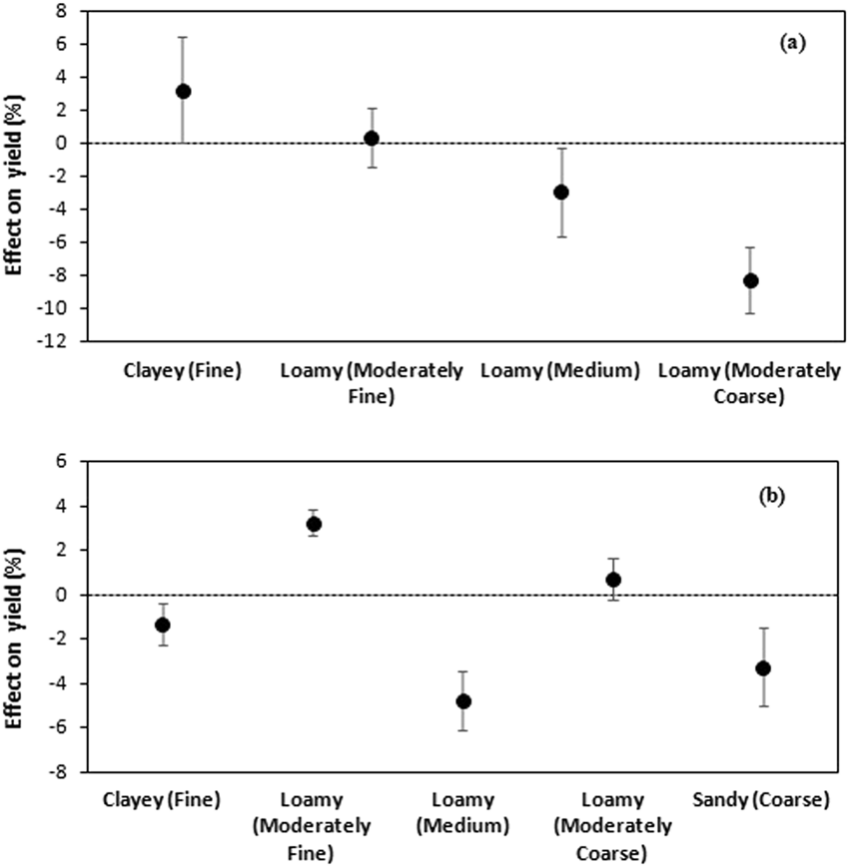
Comparison of rice grain yield in (**a**) conventional versus zero tillage irrespective of crop establishment (direct-seeding or transplanting) techniques, and in (**b**) direct-seeding versus transplanting irrespective of tillage (conventional, reduced, or zero) practices. Error bars indicate 95% confidence intervals; effect on yield is considered significant if the 95% CI does not cover zero.

The tillage (CT vs ZT) comparisons showed that grain yield responded (a) positively to ZT in the clayey group, with an average increase of 3.5%, (b) remained unchanged in moderately fine loamy soil, and (c) responded negatively in medium loamy (2.9%) and moderately coarse loamy (8.2%) groups (Fig. 2a). On the other hand, the crop establishment (TPR vs DSR) comparisons showed that grain yield of DSR responded negatively by 1.3%, 4.7%, and 3.2% in clayey, medium loamy, and sandy soils, respectively, whereas yields were higher (3.2%) in moderately fine loamy groups (Fig. 2b).

### Comparisons of water input, emissions of greenhouse gases [methane (CH_4_) and nitrous oxide (N_2_O)], cost of cultivation, and net economic returns among various tillage/CE options

**Figure 3:**
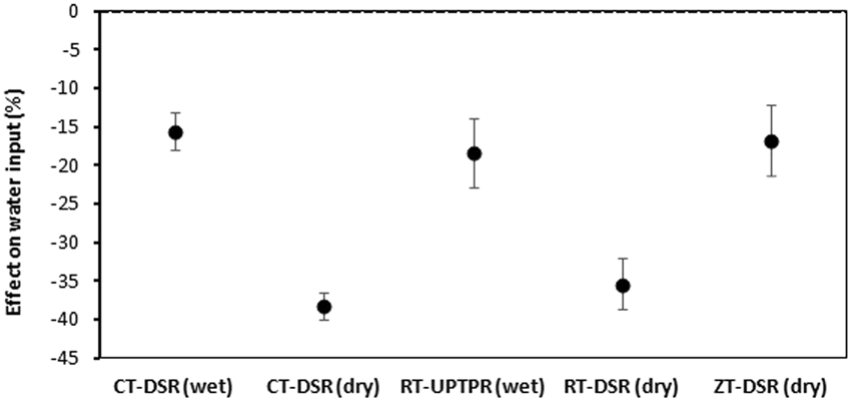
Comparison of water input in rice under five tillage/CE options compared to CT-TPR. Error bars indicate 95% confidence intervals; effect is considered significant if the 95% CI does not cover zero.

All five alternative options of tillage/CE had 15.6% to 38.3% lower water use than the control (Fig. 3). The highest savings of water took place in CT-DSR(dry), closely followed by RT-DSR(dry). Reductions in water inputs of 15.6%, 18.4%, and 16.8% in CT-DSR(wet), RT-UPTPR(wet), and ZT-DSR(dry), respectively, were comparable, whereas the decrease of 35.4% water input in RT-DSR(dry) was similar to that of CT-DSR(dry).

**Figure 4:**
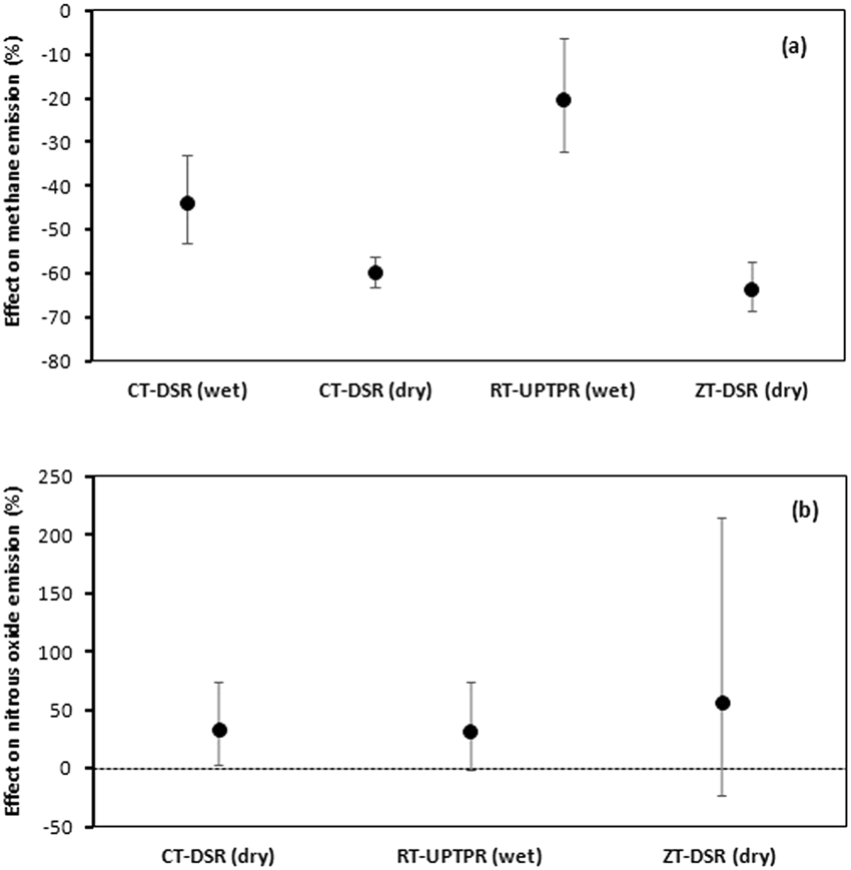
Comparison of greenhouse gas emissions: (**a**) methane and (**b**) nitrous oxide from rice fields under five tillage/CE options versus CT-TPR(wet). Error bars indicate 95% confidence intervals; effect is considered significant if the 95% CI does not cover zero.

Similar to water inputs, CH_4_ emissions were also significantly lower in four of the five tillage/CE options for which data were available than those of CT-TPR(wet), with the largest (63.4%) reduction in ZT-DSR(dry) (Fig. 4a). The reduction in CH_4_ emissions in CT-DSR(dry) (43.9%) was similar to that in ZT-DSR(dry), but was lower than in CT-DSR(wet) (59.8% less than in CT-TPR wet). The reduction in CH_4_ emissions by RT-UPTPR(wet) was comparatively less (20.1%). N_2_O emissions in meta-analysis increased by 33.7% in CT-DSR(dry) while RT-UPTPR(wet) and ZT-DSR(dry) remained unchanged (Fig. 4b).

**Figure 5:**
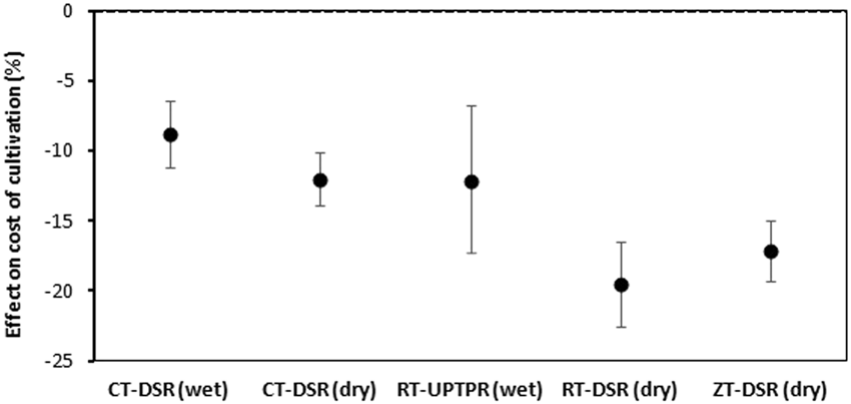
Cost of cultivation of rice under five tillage/CE options compared with CT-TPR(wet). Error bars indicate 95% confidence intervals; effect is considered significant if the 95% CI does not cover zero.

**Figure 6:**
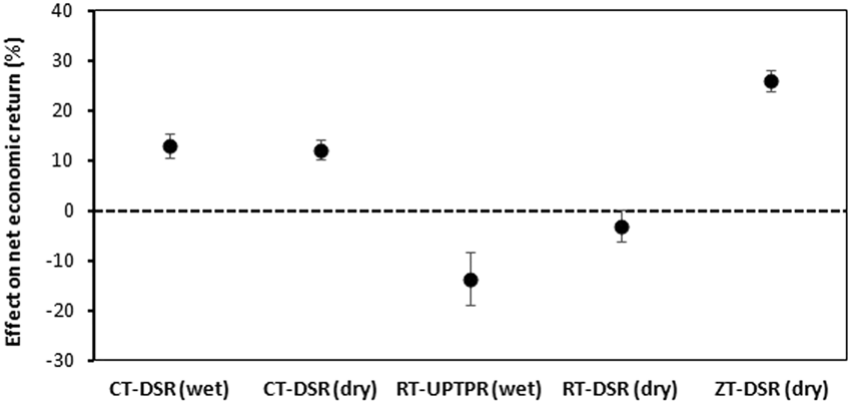
Net economic returns from rice under five tillage/CE options compared with CT-TPR(wet). Error bars indicate 95% confidence intervals; effect is considered significant if the 95% CI does not cover zero.

The analysis showed that RT-DSR(dry) and ZT-DSR(dry) had the largest reductions in the cost of cultivation, 19.6% and 17.2%, compared with CT-TPR(wet) (Fig. 5). The cost of cultivation in CT-DSR(wet), CT-DSR(dry), and RT-UPTPR(wet) was similar, ranging from 8.8% to 12.0% lower than in CT-TPR(wet). Unlike the cost of cultivation, the economic returns had a mixed trend (Fig. 6). CT-DSR(wet), CT-DSR(dry), and ZT-DSR(dry) had higher returns (13.0%, 12.1%, and 25.9%, respectively), whereas RT-UPTPR(wet) and RT-DSR(dry) had lower returns (−13.7% and −3.0%, respectively).

## Discussion

More than two-thirds of the gathered data points covered CT-DSR(wet) and CT-DSR(dry), indicating that, on a global scale, these two tillage/CE options have been predominantly evaluated and promoted. In CT-DSR(wet), substituting only the crop establishment method of labor-intensive transplanting (TPR) with that of direct-seeding (DSR) resulted in a significant yield gain. Furthermore, the combined analysis of data points from on-farm and on-station yield trials showed significantly higher yields in CT-DSR(wet). This yield gain trend comes from combined on-farm and on-station data trials, which signifies that this practice is adapted across a full spectrum of climatic and edaphic conditions. DSR (wet and dry) was estimated to occupy 21% of the total rice area of 131.6 Mha in Asia^7, 27^. The meta-analysis revealed that, in comparison with that of CT-TPR(wet), the higher yield in CT-DSR(wet) was accompanied by (a) a substantial reduction in water input by 15.6% (CI 13.1% to 18.0%), (b) a reduction in cost of cultivation by 8.8% (CI 6.4% to 11.2%), and (c) higher net economic returns by 12.1% (CI 9.3% to 15.0%). Mixed model results corroborate these findings, indicating a 1.8% (on-station + on-farm) and 4.7% (on-station) yield gain with 14.4% less water input, a reduction in cost of cultivation by 2.4%, and an increase in net return of 13%. CT-DSR(dry) yield was ‘either’ comparable with or lower than that of traditionally puddled-transplanted rice (CT-TPR wet), but had 26–38% less water input than CT-TPR(wet). CT-DSR(dry) could be a viable option for yield sustainability in the agro-ecosystems where the possibility of frequent drought occurrence is high during the growing seasons. Dry tillage is followed in CT-DSR(dry), and therefore it removes the limitation of contrasting edaphic requirements of the crops (puddled soils with continuous submergence vs well-drained soil with good tilth) when rotated in lowland (wet-puddling) and upland (dry-tillage) ecosystems^25^.

Regarding tillage, when the intensity of tillage was reduced (RT) or tillage was eliminated (ZT), a significant yield loss was observed, irrespective of whether the rice was direct-seeded or transplanted. RT-UPTPR(wet) was unsuccessful, with a significant yield penalty, implying that transplanting without puddling is an inapt option. Manual transplanting requires more labor and has higher risk of transplanting shock, which can be overcome through mechanized transplanting. Overall, our global analysis suggested that yield loss in reduced or no tillage in DSR could be less in fine clayey and moderately fine to medium loamy groups of soils where either an increase in grain yield [in RT-DSR(dry) in fine clayey soil] was estimated or the yield remained similar. On the other hand, relatively coarser textured soils might not be favorable with reduced- or no-tilled DSR, in which significant yield losses could be expected. In the northwestern Indo-Gangetic Plains (IGP) with coarse-textured soils, yield of ZT-DSR was comparable with or lower than the yield from puddle-transplanted rice^28^ or the stability of yield from ZT-DSR was poor^29^. However, reasonably good yields were obtained in the eastern IGP with fine-textured soils^30^. There were no increased economic returns in RT-DSR(dry) but ZT-DSR, despite no yield increase, attained the highest economic returns of 26–27% compared with CT-TPR(wet). This was attributed to both the yield improvement in ZT-DSR (compared to RT-DSR) and a reduction in the cost of cultivation due to savings in tillage and energy use in irrigation^26, 28, 30–32^. Avoiding soil disturbance through ZT builds soil physical condition, which also benefits the succeeding crops such as wheat or maize in rotation with rice^32–34^.

The performance of CT-DSR(wet) was better in the dry season than in the wet season, while the seasonal influence on other key tillage/CE options is mostly inconclusive. There could be poor water control, especially during and/or immediately after seeding, which may have affected crop establishment in the wet season. Excess water during the wet season is likely to have negative impacts on early initial crop establishment, tillering, lodging, and pest infestation^35, 36^. A comparable yield advantage in the dry season, on the other hand, may also be due to abundant and uninterrupted bright sunshine hour throughout the season, especially during panicle initiation to maturity^37^.The wet season is likely to induce greater plant density and higher biomass, and the cloudy days during grain formation reduce radiation sufficiently to affect the flux of assimilates for grain filling, resulting in lower yield^38^. Wet-seeded rice receives an extended window of the condition of excess water compared with transplanted rice. Our analysis is also inconclusive on yield advantage in the wet season in any of the tillage/CE options.

Although there was mixed yield response to the soil texture-groups, CT-DSR(wet) appears to be most suitable in loamy (moderately fine or moderately coarse) groups of soils. Loamy soils have better drainage than relatively fine clayey soils, and therefore offer adequate aeration. The better performance of CT-DSR(wet) in these soils is also because of less cracking of soil in the early state, which is critical for wet-seeded rice. Cracking is high in clayey soil, which directly affects the plant. The plant-available water content is also higher in these soils. Moderately fine soil may be the most favorable for CT-DSR(dry) also owing to a balance between ponding and drainage. However, water availability may emerge as a constraint in relatively coarser soils, resulting in an adverse effect on yield as revealed by our analysis.

Rice fields are the most important source of CH_4_ (a potent GHG) emissions^39–41^. CT-DSR(dry) has the potential of drastically reducing CH_4_ emissions by 33–60%, though it is known to create conditions for the emission of N_2_O^42–44^. Unlike waterlogging conditions, direct-seeded rice soils favor CH_4_ oxidation due to high soil Eh, which prevents methanogenesis^45^, and does not allow the plant-mediated transport of CH_4_ to the atmosphere^42^. Although simultaneous measurements of CH_4_ and N_2_O emissions are limited except in a few studies^42, 46^, estimations of global warming potential (GWP) in totality are rarely found. We found some N_2_O increase in CT-DSR(dry) (in RT-UPTPR(wet) and ZT-DSR(dry), it either increased or remained unchanged). However, CH_4_ emissions had a large reduction in all these tillage/CE options and, therefore, the net GWP could be potentially lower^47^. The increased GWP due to an increase in N_2_O emissions could be less than its reduction through less CH_4_ emissions, provided excess fertilizer-N is not applied^40^. Using meta-analysis, Linquist *et al*.^39^ estimated that, on average, 89% of the total GWP in rice was contributed by CH_4_. Hence, a management practice that brings a change in CH_4_ is fundamentally important to qualify as a better option. Fertilizer application, although it triggers N_2_O flux, does not alter yield-scaled GWP^42, 48^, indicating that higher yields do not necessarily bring about larger environmental footprints. The GWP estimates were lower in CT-DSR(dry) than in CT-TPR(wet)^46, 49, 50^. There is enough scope for improving global rice production to meet the demand of rice in the future^51^, although climate warming may adversely affect rice yields^52–54^. Higher CO_2_ in the atmosphere may trigger further CH_4_ emissions^55^ and the combined effect of lower yield and larger CH_4_ emissions might double the CH_4_ emissions per unit of rice yield^56^. This necessitates a better tillage and CE method to increase yield and lessen emissions so that yield-scaled GWP is reduced. It can be noted that GWP is guided by cultivar-environment-management interactions and, hence, developing a high-yielding variety adapted to an improved tillage/CE option with low GWP should be a future priority^57^. It may also be noted that a reduction in GWP through crop, water, and fertilizer management in DSR has been extensively documented in the U.S. and South America^47, 57–59^. The yield potential of DSR is very high and yield-scaled GWP is low in these regions, indicating DSR as both an economically and environmentally viable option.

In a recent study carried out in four locations in South Asia, Ladha *et al*.^26^ reported lower GWP in ZT-DSR(dry) than in CT-TPR(wet). The relatively greater reduction in CH_4_ emissions in ZT-DSR(dry) could be due to the elimination of tillage. No tillage is known to increase soil bulk density and reduce soil pore spaces, thereby likely resulting in a drastic reduction in the release of CH_4_ from the soil^60^. A slow decomposition of soil organic matter and subsequent short supply of available C in ZT-DSR(dry) may also retard CH_4_ emissions^60^.

In conclusions, our global analysis indicates that traditional rice transplanting, which is highly labor intensive and involves drudgery, can effectively be substituted by direct-seeding with no or little negative impact on rice grain yield but, more importantly, with significant reductions in water input use and methane emissions, and higher net income. Results suggest that, of the five tillage/CE options, ZT-DSR(dry), which is still in the experimental stage, appears to have the largest potential. The current meta-analysis showed that the rice yield reduction in ZT-DSR(dry) vis-à-vis CT-DSR(wet) was substantially lower (−3.0%) in on-station studies than in on-farm studies (−15.4%), indicating a need for refinement and effective extension of the technology to farmers.

Although water resources are fast depleting, farmers keep extracting groundwater at unsustainable rates because of the lack of economic incentives to minimize water use. These challenges will intensify further with projected climate change. Therefore, it is necessary to put a strategy in place to manage water resources, along with credible estimates of irrigation water use and energy requirements. Certain upland rice cultivars respond to water-saving tillage/CE options, but these cultivars are not suited to lowland areas, and give poor yields. Although significant advances have been made in agronomic aspects, there is an urgent need for rice varieties that are bred and selected for direct-seeding with no tillage^10^. It is also imperative that an effective fertilizer management strategy be evolved to minimize yield-scaled GWP. Notably, the global analysis showing that tillage/CE options have a relatively greater positive impact (a) in the dry season (potential challenge of water) than in the wet season and (b) in fine clay to moderately fine loamy soils than in coarse-textured soils highlights a need for better environmental targeting for adopting these practices. It is imperative that methods other than the conventional practice be invoked, keeping in view the dwindling natural resources and human capital participation in rice cultivation. The few tillage/CE options identified in this analysis may have potential to successfully replace the traditional method of rice establishment and cultivation. In addition, this would help national governments in formulating policies and developing plans for sustainable rice production systems and contributing to the Sustainable Development Goals.

### Limitations

A few limitations of the study can be listed. The effect of soil texture on the performance of alternative tillage/CE options is not conclusive, and the yield differences from the traditional practice vary somewhat between −14.7 and + 14.2%. Our analysis was based on published and unpublished (through personal communication) data, which do not necessarily come from experiments in which soil texture was included as a testable variable. This may be an intrinsic issue with meta-analysis of a global dataset, where one is constrained to select the studies that meet a few major criteria. Soil texture is a major factor affecting yield, primarily due to variations in water and nutrient dynamics, the latter being strongly connected to irrigation management. In flooded soil, the effect of texture becomes minimized compared with unsaturated conditions in which the texture primarily controls water-nutrient dynamics. Additionally, the irrigation management in direct-seeded rice could be sufficiently different from that of puddled-transplanted rice. We could not include studies from the North American and Latin American regions where CT-TPR(wet) as a control is not reported.

## Methods

### Data collection

Five key options of tillage/crop establishment (CE) practices (treatments) that have been evaluated extensively by researchers and farmers as potential alternatives to conventional intensive wet-tillage (puddling) and manual transplanting [CT-TPR(wet)] were selected for both meta-analysis and mixed model analysis: (1) direct-seeded rice (DSR) with conventional tillage (CT) in wet conditions [CT-DSR(wet)], (2) DSR with CT in dry conditions [CT-DSR(dry)], (3) unpuddled-transplanted rice with reduced tillage [RT-UPTPR(wet)], (4) DSR with reduced tillage in dry conditions [RT-DSR(dry)], and (5) DSR with zero tillage in dry conditions [(ZT-DSR(dry)]. The details of different tillage/CE options are given in Supplementary Table S1. CT-TPR(wet) was used as the control to compare the performance of each of the five options (pair comparisons). The data used in this study were obtained from on-station trials conducted by the researchers (a total of 3482 paired data points from 323 on-station studies) and from those conducted on-farm by the farmers in participation with the researchers (a total of 396 paired data from 9 on-farm studies) throughout the rice-growing world (Supplementary Fig. S1, Supplementary Tables S2 and S3). The on-station data were archived from 205 original peer-reviewed publications and 118 proceedings/book chapters/reports/bulletins/newsletters through the Web of Science and Google Scholar search, libraries, and personal communications (Listed in Supplementary Information). The on-farm data (unpublished) were obtained from researchers on a personal contact basis (Supplementary Table S4). Studies that met the following criteria were included only:

1. Tillage/CE practices that fall within the broad categories of five key options.
2. CT-TPR(wet) was included as a control.
3. The same location and soil type in CT-TPR(wet) and other tillage/CE options.
4. The same crop management, including fertilizer management in CT-TPR(wet) and other tillage/CE options within a study excepting treatment-specific changes such as weed and water management involved.
5. The same performance parameters and their methods of measurements in CT-TPR(wet) and other tillage/CE options within a study.

### Performance parameters and the method of measurements

The following performance parameters were considered in the analysis: (a) grain yield (Mg ha^−1^), (b) water (irrigation plus rain) input (mm ha^−1^) to the crop from sowing to harvest, (c) greenhouse (CH_4_ and N_2_O; CH_4_-C kg ha^−1^and N_2_O-N kg ha^−1^) gas (GHG) emissions, and (d) cost of cultivation and net economic returns (US$ ha^−1^) during a cropping season. Grain yield measurements were taken at harvest or at physiological maturity, and were based on crop cutting of an area ranging from 1.0 m^2^ to 100.0 m^2^ and moisture adjustment at 13–14% (alternatively, grains were oven-dried at 60–65 °C and dry weights were taken). Water input was the total amount of water supplied to the crop (rainfall plus irrigation received). Irrigation water input was computed by multiplying the discharge (measured through v-notch/parshall flume/water meter) and the time required for the irrigation. Otherwise, depth of irrigation was used to calculate the total amount of water applied to a plot. Rainfall data were measured *in situ* (by using a rain-gauge) or collected from a near-by weather station. The cost of cultivation included cost of inputs (seed, fertilizer, and other agro-chemicals used to control pests and diseases) and the cost of field operations (human labor or machines for land preparation, harvesting, and threshing and other operations, e.g., irrigation, weeding, fertilizer and agro-chemical applications, etc.). The cost of machines was calculated as consumption of diesel to run the machine for the stipulated time period multiplied by the market cost of diesel or it was directly computed from custom-hiring charges by local service providers as per local markets. Net economic returns were the difference between gross returns (obtained from the produce: multiplying grain yield by the standard price used locally in the study area; sometimes by-product (rice straw) values were also considered) and the total cost of production. All these were expressed in US$ ha^−1^ (where currency other than US$ was mentioned, it was converted to US$ with the average exchange rates of 2016). The GHG measurements were based on closed static chamber techniques with gas samples analyzed using a gas chromatograph equipped with a flame ionization detector (for CH_4_) and an electron capture detector (for N_2_O). Emissions between two adjacent measurement-days were interpolated and total gas emissions over the season were cumulated. There were only a few GHG studies in which (a) all five CE/tillage options along with the respective control [CT-TPR(wet)] were evaluated, and (b) all the performance parameters considered in the study were measured. No studies with RT-DSR(dry) had CH_4_ and N_2_O measurements, and studies with CT-DSR(wet) lacked N_2_O measurements.

### Categorical variables

The performance parameters were further categorized based on soil texture, rice-growing season, the magnitude of tillage, and crop establishment method. Soil textural classes of the study areas were categorized into five texture-groups^61^: clayey (fine), loamy (moderately fine, medium, moderately coarse), and sandy (coarse). Rice is mainly grown in the rainy season (July to November), referred to as the wet season (WS), which is primarily rainfed but with need-based supplemental irrigation. In some areas, rice is also grown in the dry season (DS; January to May), which is primarily irrigated. Three broad categories of tillage (conventional, reduced, and zero or no tillage) and two categories of crop establishment (transplanting and direct-seeding) were also used. Since the datasets of all the performance parameters were available only in the studies conducted at experimental stations, the on-station data were used for the comparisons. In addition, meta-analysis and mixed model analysis of combined (on-station plus on-farm) datasets of grain yield were also done to investigate the differences in performance between on-station and on-farm studies. To investigate the effect of (a) tillage (CT vs ZT), the data points of options 1 and 2 were combined to form the CT group and options 4 and 5 were combined to form the RT/ZT group [no data were available for RT/ZT combination in sandy (coarse) group], and (b) to investigate crop establishment (TPR vs DSR), the data points of CT-TPR(wet) and RT-UPTPR(wet) were used as the group of TPR and the data points of options 1, 2, 4, and 5 were combined to form the DSR group. In addition, other basic parameters such as location, key soil characteristics, fertilizer inputs, sowing and harvest time, seed rate, and weed control were recorded.

### Statistical analysis

All the variables were subjected to (a) meta-analysis and (b) mixed model analysis. Meta-analysis has attracted considerable attention recently as a powerful tool to analyze the response of treatments to controls from diverse individual studies to evolve to a general global trend or pattern. Mixed model analysis allows a wide variety of correlation (variance-covariance) structure and residual error variance between studies to be explicitly modeled. Our data come from a wide variation of soil, agroclimatic conditions, and management practices, for which the mixed model offers the most flexibility in handling such heterogeneous datasets.

#### Meta-analysis

The meta-analysis was performed by using MetaWin 2.1 in two stages^62^. At first, the effect size was calculated for each study as the natural log of the response ratio (lnR) using the following equation^63^:

1 $${\rm{Effect}}\,{\rm{size}}=\,\mathrm{ln}\,{\rm{R}}=\frac{{{\rm{X}}}_{({\rm{TCE}})}}{{{\rm{X}}}_{({\rm{CT}}-{\rm{TPR}})}}$$

where X_TCE_ is the mean of response variables (yield, water input, GHG emissions, cost of cultivation, and net economic returns) of the five tillage/CE options, and X_CT-TPR_ is the mean of these variables in CT-TPR(wet). Since most studies did not report the variance of the means of response variables, the effect sizes were weighted based on the number of replicates as follows^64^:

2 $$Weight=\frac{{n}_{(TCE)}\times {n}_{(CT-TPR)}}{{n}_{(TCE)}+{n}_{(CT-TPR)}}$$

where ${n}_{({TCE})}$ and ${n}_{({CT}-{TPR})}$ represent the number of replications for each of the five key tillage/CE options and CT-TPR(wet), respectively, in an individual study. If more than one observation was included in an option, the weights were divided by the number of observations from that study^65^. Extreme outliers were identified as more than 3*standard deviations from the weighted mean effect size within each category and were removed.

Effect sizes from individual studies were then combined using a mixed-effect model to calculate the cumulative effect size and the 95% confidence intervals (CIs) through bootstrapping with 4999 iterations^64^. The mixed-effect model is a random-effect meta-analytic model for categorical data^62^, assuming random variation among studies within a group and fixed variation between groups. The cumulative effect was considered significant if the CIs did not overlap with zero. Effect sizes among the categories were considered significantly different if their CIs did not overlap. For ease of interpretation, results were back-transformed and reported as percentage change caused by tillage/CE options in relation to CT-TPR(wet). All the results were discussed as the change over CT-TPR(wet) (the control), and only the significant changes were analyzed. Unless stated otherwise, differences were considered significant only when p values were <0.05.

#### Mixed model analysis

The data were subjected to mixed model analysis to estimate the effects of the tillage and CE options over CT-TPR(wet) as the control, and to test the differences among them (i) for yield (Mgha^−1^), total water input (mm ha^−1^), CH_4_ and N_2_O emissions (CH_4_-C kg ha^−1^ and N_2_O-N kg ha^−1^, respectively), cost of cultivation, and net economic returns (US$ ha^−1^) across all studies included in the analysis. Mixed model analysis was also performed for yield estimates to quantify differences among (i) wet and dry seasons, (ii) transplanted and direct-seeded conditions, and (iii) different soil textures. The analysis was carried out using the MIXED procedure of SAS^66^ taking the different tillage/CE options, including the control (CT-TPR), as fixed effects and the studies as random effects. Least squares means were compared using the algorithm for a compact letter display of comparisons for unbalanced data^67^.

Generally, the meta- and mixed model analyses showed similar trends and, therefore, mostly the meta-analysis results are described in greater detail. Many of the mixed model outputs are provided in the Supplementary Information while comparing with the meta-analysis. In the discussion, however, inferences were drawn from both the meta-analysis and mixed model results.

## Electronic supplementary material

Supplementary Information
