# Supplementary material for: A global analysis of alternative tillage and crop establishment practices for economically and environmentally efficient rice production
Source: Sci Rep. 2017 Aug 24;7:9342. doi: 10.1038/s41598-017-09742-9 (PMC5571152; doi:10.1038/s41598-017-09742-9)
Supplement: Supplementary file 1 — Supplementary Information [file 41598_2017_9742_MOESM1_ESM.pdf]

Supplementary Information

A global analysis of alternative tillage and crop establishment practices for economically and environmentally efficient rice production

Debashis Chakraborty[1], Jagdish Kumar Ladha[2,*], Dharamvir Singh Rana[2], Mangi Lal Jat[3], Mahesh Kumar Gathala[3], Sudhir-Yadav[2], Adusumuli Narayan Rao[5], Mugadoli S. Ramesha[2], and Anitha Raman[2]

[1]ICAR-Indian Agricultural Research Institute, New Delhi, 110012, India.
[2]International Rice Research Institute, Las Baños, Philippines.
[3]International Maize and Wheat Improvement Center-Bangladesh, Dhaka, 1212, Bangladesh.
[4]International Maize and Wheat Improvement Center, New Delhi, 110012, India.
[5]International Crops Research Institute for Semi Arid Tropics, Patancheru, 500033, India.

*Corresponding author (email: j.k.ladha@irri.org).

A global analysis of alternative tillage and crop establishment practices for economically and environmentally efficient rice production

Mixed model analysis

Comparisons of grain yield among various tillage and CE options (a) on-station vs on-farm and (b) in dry season vs wet season. A significant increase in yield was obtained under CT-DSR(wet) by 0.20 and 0.17 Mg ha^{-1} vis-à-vis a yield of 4.87 Mg ha^{-1} in CT-TPR(wet) in both the dry and wet season, respectively (Supplementary Table S5). The remaining tillage/CE options had lower yield than CT-TPR(wet) in both seasons.

Comparisons of grain yield among various tillage/CE options in response to soil texture: (a) conventional tillage vs zero tillage and (b) transplanting vs direct-seeding. The range of yield response to tillage/CE options varied from -0.52 to + 1.13 Mg ha^{-1} (corresponding to -23.5% to +15.1% of the yield estimates in CT-TPR(wet) (Supplementary Table S6). The performance of CT-DSR(wet) in medium- to coarse-textured soil groups tends to be superior, with +0.30 to +0.52 Mg ha^{-1} yield gains. Yield in CT-DSR(dry) declined in fine clayey (-0.37 Mg ha^{-1}) and moderately fine to medium loamy soils (-0.60 and -0.28 Mg ha^{-1}), but was higher than in CT-TPR(wet) in moderately coarse loamy soil (+0.19 Mg ha^{-1}). RT-UPTPR(wet) yields remained unchanged in both of these texture groups, and both RT-DSR(dry) and ZT-DSR(dry) could be potentially favorable options in medium and moderately coarse loamy soils with no yield difference in other texture groups.

A comparison of CT and ZT using mixed model revealed no significant yield differences in clayey and moderately fine loamy soils, but a gradually increasing yield reduction under RT/ZT as the texture becomes coarser (-0.33, -0.41, and -0.78 Mg ha^{-1} in loamy-medium, loamy-moderately coarse, and sandy-coarse soils, respectively) (Supplementary Table S7). A comparison among crop establishment methods showed a yield reduction in DSR in clayey, medium loamy, and moderately coarse loamy soils (-0.14 to -0.41 Mg ha^{-1}), a gain in moderately fine loamy soils (+0.24 Mg ha^{-1}), and no significant change in yield in sandy coarse soils (Supplementary Table S7).

Comparisons of water input, emissions of greenhouse gases (methane, nitrous oxide), cost of cultivation, and economic returns among various tillage/CE options. Compared with CT-TPR(wet), all five tillage/CE options required 1371 to 1710 mm ha^{-1} (8.0% to 26.3%) lower total (rain + irrigation) water inputs (Supplementary Table S8). The maximum reduction was in CT-DSR(dry) (-489 mm ha^{-1}), followed by RT-DSR(dry) (375 mm ha^{-1}). Savings of water were comparable in CT-DSR(wet), RT-UPTPR(wet), and ZT-DSR(dry), respectively.

CT-DSR(dry), RT-UPTPR(wet), and ZT-DSR(dry) contributed 64, 73, and 74 kg CH_4-C ha^{-1} emissions, respectively, which were significantly lower than CT-TPR(wet) (96 kg of CH_4-C ha^{-1}). These corresponded to 22.9% to 33.3% reductions in CH_4 emissions. On the contrary, there was a 50% increase in NO_2-N emissions from ZT-DSR(dry) compared with CT-TPR(wet)]. No significant changes were observed in NO_2-N emissions in either CT-DSR(dry) or RT-UPTPR(wet) (Supplementary Table S8).

The cost of cultivation was significantly lower in CT-DSR(dry), RT-DSR(dry), and ZT-DSR(dry), ranging from US$322 to 402 ha^{-1} [2.4-21.8% less than CT-TPR(wet)] (Supplementary Table S8). The cost incurred for CT-DSR(wet) was similar to that for CT-TPR(wet), but the net return was higher by US$49 ha^{-1} [13% more than CT-TPR(wet)]. Returns were higher also in CT-DSR(dry) and ZT-DSR(dry) (17.9% and 27.5%, respectively) than in CT-TPR(wet). RT-UPTPR (wet) did not differ significantly from CT-TPR(wet).

The Supplementary Table S9 gives a comparative summary of the meta-analysis and mixed model outputs of tillage/CE options on yield and other parameters, over the conventional practice [CT-TPR(wet)], and the same has been described at appropriate places.

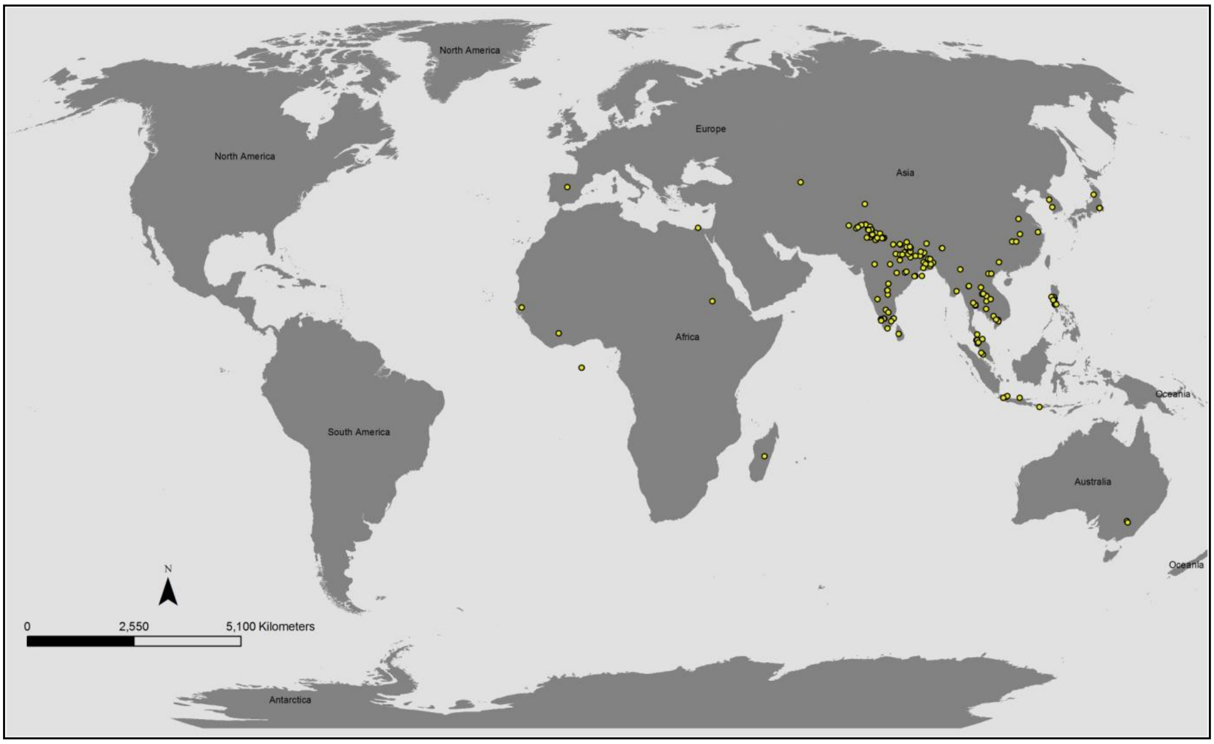

Supplementary Figure S1. Map showing the locations of 323 on-station studies. Studies from the U.S. and South America could not be considered in our analysis due to non-availability of CT-TPR(wet) as the control; Prepared by using Q-GIS ver. 2.18 [Quantum GIS Development Team 2009. QGIS Geographic Information System. Open Source Geospatial Foundation. URL http://qgis.osgeo.org]

Supplementary Table S1. Details of the conventional method of rice cultivation and the five potential tillage and crop establishment alternatives adopted in the meta-analysis

Name	Abbreviation	Tillage	Crop establishment method
Conventionally tilled (CT) under wet conditions and transplanting of rice seedlings (TPR)	CT-TPR(wet)	Two to three dry harrowings are done followed by flooding and two to three wet-tillage (puddling) operations. The land was levelled by one to two plankings.	Two- to 4-week-old seedlings are transplanted manually in rows on the puddled soil; full saturation or standing water (2-5 cm) is maintained till 1-2 weeks prior to harvest. Normally, continuous submergence (5-10 cm water) is maintained from transplanting until maturity or 1-2 weeks before harvest.
Conventionally tilled (CT) under wet conditions and direct-seeding of rice (DSR)	CT-DSR(wet)	Same as CT-TPR (wet)	Pre-germinated seeds (soaked for 24-48 h in water, covered and drained for 36-72 h)are broadcast manually, sown in line manually (or using a drum seeder) in well-puddled seedbeds, or sown into pre-standing water or saturated soil.Normally, continuous submergence (5-10 cm water) is maintained from seeding until maturity or 1-2 weeks before harvest.
CT under dry conditions and DSR	CT-DSR(dry)	Land is plowed in dry conditions (2-3 passes) by using a cultivator, rotavator, or disc, followed by levelling the field by wooden planker.	Dry or primed (water-soaked for 24 h and incubated) seeds are broadcast, drilled, or sown in furrows behind the plow and dibbling, followed by irrigation to flooding or keeping the soil wet for about 1 week to ensure germination.<br>Normally, rice is rainfed (favorable) with supplementary irrigation to avoid water stress.
Reduced tillage (RT) and transplanting of rice under non-puddled (UPTPR) conditions	RT-UPTPR(wet)	Normally, land is shallow-tilled with a single pass or strip tillage under wet conditions and is leveled. Occasionally, raised beds (37 cm wide, 15 cm high, and 30-cm furrows in between) arealso formed.	Rice is transplanted using 2-4-week-old seedlings in 20x20-cm spacing (or two rows with 20-cm spacing on raised beds) in moist conditions. Normally, continuous submergence (5-10 cm water) is maintained from seeding until maturity or 1-2 weeks before harvest. In raised beds, water is maintained in furrows.

RT under dry conditions and DSR	RT-DSR(dry)	Normally, land is shallow tilled with a single pass or strip tillage under dry conditions and levelled. Occasionally, raised beds (37 cm wide, 15 cm high, and 30-cm furrows in between) are also formed.	Dry pre-germinated seeds are sown using a drill on tilled flat land or on raised beds (two rows with 20-cm spacing on raised beds). Normally, it is rainfed (with occasional supplemental irrigation) where the water regime may vary from dry to occasional flooding. In raised beds, water is maintained in furrows.
Zero tillage and DSR	ZT-DSR(dry)	No tillage (only opening of slits for seed placement)	Dry seeds are drill-sown or sown in slits opened by a zero-till drill followed by irrigation for proper seed germination. Normally, irrigation with the appearance of hairline cracks in the soil surface and occasional flooding.

Supplementary Table S2. Number of data points and number of studies (in parentheses) for various performance parameters included in the analysis breakup of yield data points into five texture groups under different tillage and CE options

Tillage and CE options*	Parameter						
	Yield		Total water input	Cost	Net economic return	CH_4 emissions	N_2O emissions
	On-station	On-station + on-farm	On-station				
CT-DSR(wet)	1253 (168)	1314 (229)	102 (27)	51 (24)	75 (28)	8 (3)	-
CT-DSR(dry)	1827 (197)	1956 (266)	109 (28)	74 (23)	99 (31)	34 (14)	47 (7)
RT-UPTPR(wet)	96 (29)	146 (59)	30 (12)	12 (7)	21 (11)	9 (6)	6 (3)
RT-DSR(dry)	97 (39)	155 (62)	35 (14)	26 (9)	28 (10)	-	-
ZT-DSR(dry)	209 (49)	307 (103)	30 (11)	54 (16)	78 (20)	12 (5)	10 (3)

*Refer Supplementary Table S1 for details

Supplementary Table S3. Number of data points and number of studies (in parentheses) of different texture groups included in the analysis

Tillage and CE options*	Soil texture groups				
	Clayey	Loamy			Sandy
	Fine	Moderately fine	Medium	Moderately coarse	coarse
CT-DSR(wet)	308 (26)	603 (89)	94 (19)	214 (34)	34 (5)
CT-DSR(dry)	211 (24)	909 (90)	219 (32)	367 (48)	121 (14)
RT-UPTPR(wet)	18 (2)	30 (12)	13 (5)	35 (14)	-
RT-DSR(dry)	11 (3)	15 (12)	22 (9)	49 (18)	-
ZT-DSR(dry)	26 (5)	97 (24)	35 (9)	51 (17)	-

*Refer Supplementary Table S1 for details

Supplementary Table S4. On-farm data inventory of rice grain yield: Details

S. no.	Country	District	Year of trial	Treatment	Number of data points
1	Bangladesh	Comilla	2011, 2013	T3,T4,T5,T6[#]	252
		Rajshahi	2011, 2012	T3,T4,T5,T6	318
		Mithapukur	2010, 2011	T2,T4	26
		Rangpur	2011, 2012, 2013	T3,T4,T5,T6	180
		Gangachara	2009, 2010, 2011	T2,T3,T4,T5,T6	55
2	India	Ballia	2007, 2009, 2010	T3,T4,T5,T6	396
		Begusarai	2009, 2011	T3,T4,T6	139
		Ghazipur	2010	T3,T4,T6	22
		Gorakhpur	2010	T3	4
		Jamui	2009, 2010	T3,T5,T6	44
		Kaithal	2011	T3	7
		Karnal	2007, 2011	T3,T5	81
		Katihar	2009, 2010	T6	57
		Lakhisarai	2009, 2010, 2011	T3,T4,T5,T6	282
		Mahrajganj	2010	T3,T4	92
		Meerut	2007	T5	79
		Muzaffarpur	2009, 2010	T6	97
		Panipat	2011	T3	40
		Patna	2009, 2010, 2011	T3,T4,T6	132
		Purnea	2010	T6	55
		Samastipur	2009, 2011, 2012	T3,T4,T5,T6	122
		Sonepat	2011	T3	8
		Vaishali	2009, 2010, 2011	T6	34
		Raichur	2013, 2014	T2,T3	246
		Thanjavur	2011	T5	146
		Yadgir	2014	T3	83
		Faridkot	2012	T3	135
		Moga	2012	T3	10
		Nawada	2010	T4,T6	17
		Ropar	2010, 2012	T3	88
3	Nepal	Bara	2009, 2010	T2,T4	71
		Chitwan	2010	T2,T3,T4,T5	140
		Nawalparasi	2010	T2	44
		Parsa	2009, 2010	T2,T4	37
		Rupandehi	2009, 2010	T2,T4	95

T2: CT-DSR(wet); T3: CT-DSR(dry); T4: RT-UPTPR(wet); T5: RT-DSR(dry); T6: ZT-DSR(dry)

Supplementary Table S5. Mixed model estimation of seasonal variation in rice yield (Mg ha^{-1}) under CT-TPR(wet) and tillage/CE options

Tillage and CE options*	Season	
	Dry	Wet
CT-TPR(wet)	4.87(0.091)c	4.87(0.098)c
CT-DSR(wet)	5.07(0.095)d	5.03(1.03)d
CT-DSR(dry)	4.75(0.092)b	4.77(0.099)b
RT-UPTPR(wet)	4.60(0.150)b	4.66 (1.64)bc
RT-DSR(dry)	4.26(0.146)a	4.27 (0.153)a
ZT-DSR(dry)	4.79(0.122)bc	4.80 (0.129)bc

Standard errors are given in parentheses; means followed by the same letters are not significantly different at $p<0.05$.

*Refer Supplementary Table S1 for details

Supplementary Table S6. Mixed model estimates of rice grain yield (Mg ha^{-1}) in CT-TPR(wet) and five tillage and CE options under five soil texture-groups

Tillage and CE options#	Texture-groups				
	Clayey (fine)	Loamy (moderately fine)	Loamy (medium)	Loamy (moderately coarse)	Sandy (coarse)
CT-TPR(wet)	4.59(0.199)	4.80(0.159)	4.94(0.134)	4.89(0.110)	5.12 (0.359)
CT-DSR(wet)	4.64(0.203)*	4.60(0.199)**	5.24 (0.160)*	5.22 (0.116)**	5.64 (0.332)*
CT-DSR(dry)	4.22 (0.197)*	4.20 (0.161)**	4.66(0.137)**	5.09(0.112)**	4.63 (0.352)
RT-UPTPR(wet)	3.92 (0.371)	4.28(0.324)	4.68 (0.216)	5.02(0.221)	4.98(1.139)
RT-DSR(dry)	3.92(0.385)	3.85 (0.265)*	4.36(0.195)**	4.61(0.303)	-
ZT-DSR(dry)	4.84 (0.303)	4.45(0.231)*	4.60(0.194)*	5.12 (0.164)	3.98(1.138)

Values in parentheses are standard errors; * and **indicate significant differences ($p<0.05$ and $p<0.001$, respectively). #Refer Supplementary Table S1 for details

Supplementary Table S7. Mixed model estimates of rice grain yield (Mg ha^{-1}) in conventional (CT) vs zero (ZT) tillage systems (irrespective of crop establishment methods) and in direct-seeded (DSR) vs transplanting (TPR) (irrespective of tillage practices) under different soil texture-groups

Tillage and CE options*	Texture-groups				
	Clayey (fine)	Loamy (moderately fine)	Loamy (medium)	Loamy (moderately coarse)	Sandy (coarse)
(a) Tillage					
CT	5.29 (1.076)	5.44 (0.356)	5.27(0.343)	5.10 (0.242)	4.56(0.520)
RT/ZT	5.21 (1.078)	5.41 (0.357)	4.94 (0.351)	4.69(0.243)	3.78 (0.541)
p value	*0.839*	*0.845*	*0.044*	*<0.001*	*0.033*
(b) Crop establishment					
DSR	4.87(0.291)	5.09(0.127)	4.51 (0.182)	4.54(0.186)	4.60(0.408)
TPR	5.28(0.299)	4.85(0.130)	4.73(0.188)	4.69 (0.190)	4.71(0.420)
p value	*0.0002*	*<0.001*	*0.009*	*0.023*	*0.515*

The numbers in parentheses are the standard errors of the mean.
*Refer Supplementary Table S1 for details

Supplementary Table S8. Mixed model estimates of total water input, greenhouse gas (CH_4 and N_2O) emissions, cost of cultivation, and net economic returns in CT-TPR(wet) and five tillage/CE options.

Tillage and CE options*	Total water input	CH_4 emissions	N_2O emissions	Cost of cultivation	Net economic returns
	mm ha^{-1}	kg CH_4-C ha^{-1}	kg N_2O-N ha^{-1}	US$ ha^{-1}	
CT-TPR(wet)	1860(103)d	96(30)b	1.34(0.41)a	412d	375(32)a
CT-DSR(wet)	1591(108)bc	-	-	402cd	424(37)bc
CT-DSR(dry)	1371(107)a	64(30)a	1.57(0.43)ab	378bc	442(36)bc
RT-UPTPR(wet)	1710(123)c	73(30)a	1.76(0.55)ab	407cd	398(48)ac
RT-DSR(dry)	1485(119)ab	-	-	322a	377(45)ab
ZT-DSR(dry)	1582(122)bc	74(30)a	2.01(0.51)b	354b	478(37)c

Estimates are followed by standard errors in parentheses. Values with the same letters in a column are not significantly different at $p<0.05$.
The numbers in parentheses are the standard errors of the mean. Means followed by the same letters are not significantly different (at 5%).
*Refer Supplementary Table S1 for details

Supplementary Table S9. A comparative summary of the meta and mixed model analyses of tillage and CE options on yield and other parameters, over the conventional puddle transplanting of rice [CT-TPR(wet)]; (+), (-) and (0) refer to significantly positive, negative and no change, respectively. NA denotes no data available.

Tillage and CE options	Parameters											
	Yield		Total water input		Cost of cultivation		Net economic return		CH_4 emission		N_2O emission	
	Meta	Mixed	Meta	Mixed	Meta	Mixed	Meta	Mixed	Meta	Mixed	Meta	Mixed
CT-DSR(wet)	(+)	(+)	(-)	(-)	(-)	(0)	(+)	(+)	(-)	NA	NA	NA
CT-DSR(dry)	(0)	(-)	(-)	(-)	(-)	(-)	(+)	(+)	(-)	(-)	(+)	(0)
RT-UPTPR(wet)	(-)	(0)	(-)	(-)	(-)	(0)	(-)	(0)	(-)	(-)	(0)	(0)
RT-DSR(dry)	(-)	(-)	(-)	(-)	(-)	(-)	(0)	(0)	NA	NA	NA	(-)
ZT-DSR(dry)	(-)	(0)	(-)	(-)	(-)	(-)	(+)	(+)	(-)	(-)	(0)	(+)

List of on-station studies included in the analysis

1. Rashid, M.H., Alam, M.M., Khan, M.A.H. & Ladha, J.K. Productivity and resource use of direct-(drum)-seeded and transplanted rice in puddled soils in rice-rice and rice-wheat ecosystem. *Field Crops Res*. **113**, 274–281 (2009).
2. Kato, Y., Okami, M. & Katsura, K. Yield potential and water use efficiency of aerobic rice (*Oryza sativa* L.) in Japan. *Field Crops Res*. **113**, 328–334 (2009).
3. Bazaya,B.R., Sen,A. & Srivastava,V.K. Planting methods and nitrogen effects on crop yield and soil quality under direct-seeded rice in the Indo-Gangetic plains of eastern India. *Soil Till. Res*. **105,** 27–32 (2009).
4. Jat, M.L., *et al*. Evaluation of precision land levelling and double zero-till systems in the rice-wheat rotation: Water use, productivity, profitability and soil physical properties. *Soil Till. Res*. **105,** 112–121 (2009).
5. Singh, Y., *et al*. Crop performance in permanent raised bed rice-wheat cropping system in Punjab, India. *Field Crops Res.***110,** 1–20 (2009).
6. Thakur, A.K., Roychowdhury, S., Kundu, D.K. & Singh, R.Evaluation of planting methods in irrigated rice. *Arch. Agron.Soil Sci*. **50,** 631–640 (2004).
7. Ma'shum, M., *et al*. Rice responses to soil management in a rice-based cropping system in the semi-arid tropics of southern Lombok, Eastern Indonesia. *Field Crops Res*. **110**, 197–206 (2009).
8. Dunn, B.W., Mathews, S.K., Beecher, H.G., Thompson, J.A. & Humphreys, E. Growing rice on raised beds in south-eastern Australia. In: Fischer, T., Turner, N., Angus, J., McIntyre, L., Robertson, M., Borrell, A., Lloyd, D. (eds.) New directions for a diverse planet: Proceedings for the 4th International Crop Science Congress, 26 September – 1 October, Brisbane, Australia (2004).
9. Bajpai, R.K. & Tripathi, R.P. Evaluation of non-puddling under shallow water tables and alternative tillage methods on soil and crop parameters in a rice-wheat system in Uttar Pradesh. *Soil Till. Res*. **55**, 99–106 (2000).
10. Iqbal, E., Ahmad, A. & Randhwa, S.A. Effect of direct seeding and transplanting method on the yield and quality of fine rice Basmati-370. *Int. J. Agric. Biol*. **2,** 251–252 (2000).
11. Tabbala, D.F., Bouman, B.A.M., Bhuiyana, S.I., Sibayanb, E.B. & Sattarc, M.A. On-farm strategies for reducing water input in irrigated rice; case studies in the Philippines. *Agric. Water Manage.* **56**, 93–112 (2002).
12. Arromratana, U., *et al*. Development of no-tillage direct seeding cultivation of rice. *JIRCAS Working Report*. **30**, 129–136 (2002).
13. Garcia, F.V., Peng, S. & Cassman, K.G. Yield potential of transplanted and wet seeded rice in high yield environments in the Philippines. Proc. Intl. Workshop on Constraints, opportunities and innovations for wet seeded rice, May 31-June 3, Bangkok, Thailand. 246–256 (1994).
14. Anil Prashar, S., et al. Performance of rice on beds and puddled transplanted flats in Punjab, India. © 2004. New directions for a diverse planet: *Proceedings of the 4th International Crop Science Congress, Brisbane, Australia, 26 Sep - 1 Oct. ISBN 1 920842 20 9.*www.cropscience.org.au (2004).
15. Yamauchi, M., *et al.* Seedling establishment and grain yield of tropical rice sown on puddled soil. *Agron. J*.**92,** 275–282 (2000).
16. Tang, Y., Zheng, J., Huang, G., & Du, J. Study on permanent bed planting with double zero tillage for rice and wheat in Sichuan Basin. • New directions for a diverse planet. Edited by R.A. Fischer. Proceedings of the 4th International Crop Science Congress. Brisbane, Australia, 26 September-1 October (2004).

17. Johnkutty, I., Gracy, Mathew & Jose Mathew. Comparison between transplanting and direct seeding methods for crop establishment in rice. *J. Trop. Agric.* **40,** 65–66 (2002).
18. Hobbs, P.R., Singh, Y., Giri, G.S., Lauren, J.G., & Duxbury, J.M. Direct-seeding and reduced tillage options in the rice-wheat systems of the Indo-Gangetic plains of South Asia. In: Pandey, S., Mortimer, M., Wade, L., Tuong, T.P., Lopez, K., Hardy, B., editors. 2002. Direct seeding: research issues and opportunities. Proceedings of the International Workshop on Direct Seeding in Asian Rice Systems: Strategic Research Issues and Opportunities, 25-28 January 2000, Bangkok, Thailand. Los Baños (Philippines): International Rice Research Institute. **383,** 201–215 (2002).
19. Lav Bhushan, *et al.*Saving of water and labor in a rice–wheat system with no-tillage and direct seeding technologies. *Agron. J.* **99,** 1288–1296 (2007).
20. Justo, Jr. H.D. & Rillon, G.S. Differences in pest incidence and yield between direct-seeded and transplanted rice crops. *INMnet Bull.* **4,** 14 (1998).
21. Thilagavathi, T.& Ramanathan, S. Nitrogen management for direct wet-seeded rice. *Intl. Rice Res. Notes* **30,** 44–45 (2005).
22. Subramanian, E., James Martin, G. & Ramasamy, S. Effect of weed and nitrogen management on weed control and productivity of wet seeded rice. *Indian J. Weed Sci.* 37(1 & 2), 61–64 (2005).
23. Tuong, T.P., Singh, A.K., Siopongco, J.D.L.C. & Wade, L.J. Constraints to high yield of dry-seeded rice in the rainy season of a humid tropic environment. *Plant Prod. Sci.* 3, 164–172 (2000).
24. Ikeda, H., Kamoshita, A., Yamagishi, J., Ouk, M. & Lor, B. Assessment of management of direct seeded rice production under different water conditions in Cambodia. *Paddy Water Environ.* 6, 91–103 (2008).
25. Won, J.G., Choi, C.D. & Lee, S.C. Tillering, lodging and yield under deep water treatment in direct-seeded rice. *Plant Prod. Sci.* 2, 200–205 (1999).
26. Bisht, P.S., Puniya, R., Pandey, P.C. & Singh, D.K. Grain yield and yield components of rice as influenced by different crop establishment methods. *Intl. Rice Res. Notes* 32, 33–34 (2007).
27. Bouman, B.A.M., Peng, S., Castaneda, R.M. & Visperas, R.M. Yield and water use of irrigated tropical aerobic rice systems. *Agric. Water Manage.* 74, 87–105 (2005).
28. Sattar, M.A. & Bhuiyan, S.I. Performance of direct-seeded and transplanted rice under different water management practices. *Bangladesh Rice J.* 5, 1–5 (1994).
29. Farooq, M., Basra, S.M.A. & Asad, S.A. Comparison of conventional puddling and dry tillage in rice-wheat system. *Paddy Water Environ.* 6, 397–404 (2008).
30. Naklang, K.,Fukai, S.& Nathabut, K. Growth of rice cultivars by direct seeding and transplanting under upland and lowland conditions. *Field Crops Res.* 48, 115–123 (1996).
31. Choudhury, B.U., Bouman, B.A.M. & Singh, A.K. Yield and water productivity of rice-wheat on raised beds at New Delhi, India. *Field Crops Res.* 100, 229–239 (2007).
32. Bhuiyan, S.I., Sattar, M.A. & Khan, M.A.K. Improving water use efficiency in rice irrigation through wet-seeding. *Irrig. Sci.* 16, 1–18 (1995).
33. George I. Ghobrial. Effects of level, time and splitting of urea on the yield of irrigated direct-seeded rice. *Plant Soil* 56, 209–215 (1980).
34. Peng, S., et al. Nitrogen use efficiency of irrigated tropical rice established by broadcast wet-seeding and transplanting. *Fert. Res.* 45, 123–134 (1996).
35. Stevens, G., Hefner, S., & Gladbach, T. Effect of nitrogen timing and rates on tillering and yield of no-till water-seeded rice. *Commun. Soil Sci. Plant Anal.* 32, 421–428 (2001).
36. Santhi, P., Ponnuswamy, K. & Kempuchetty, N. A labor-saving technique in direct-sown and transplanted rice. *Int. Rice Res. Notes* 23, 35–36 (1998).

37. Lee, K.S., Jun, J.H. & Shin, H.T. Effective amount of N fertiliser for direct seeding on wet surface of reclaimed saline soil in Korea. *Int. Rice Res. Notes* 23, 29 (1998).
38. Budhar, M.N. & Tamilselvan, N. Evaluation of stand establishment techniques in lowland irrigated rice. *Int. Rice Res. Notes* 26, 72–73 (2001).
39. Ando, H., Kakuda, K., Nakayama, M. & Yokoto, K. Yield of no-tillage direct-seeded lowland rice as influenced by different sources and application methods of fertiliser nitrogen. *Soil Sci. Plant Nutr.* 46, 105–115 (2000).
40. Guyer, R. & Quadranti, M. Effect of seed rate and nitrogen level on the yield of direct wet-seeded rice. In: Proc. APWSS. 304–311 (1985).
41. Sharma, S.K., Tomer, R.K. & Gangwar, K.S. Effect of crop-establishment and tillage practices on yield and economics of irrigated rice (*Oryza sativa*) - wheat (*Triticum aestivum*) system. *Indian J. Agric. Sci.* 65, 636–638 (1995).
42. Hukkeri, S.B. & Sharma, A.K. Water-use efficiency of transplanted and direct-sown rice under different water management practices. *Indian J. Agric. Sci.* 50, 240–243 (1980).
43. Singh, Y., Gupta, R.K., Singh, B. & Gupta, S. Efficiency of fertiliser nitrogen in wet direct-seeded rice (Oryza sativa) in northwest India. *Indian J. Agric. Sci.* 77, 561–564 (2007).
44. Gill, M.S., Kumar, A. & Kumar, P. Growth and yield of rice (*Oryza sativa*) cultivars under various methods and times of sowing. *Indian J. Agron.* 51, 123–127 (2006).
45. Gill, M.S., Kumar, P. & Kumar, A. Growth and yield of direct-seeded rice (*Oryza sativa*) as influenced by seeding technique and seed rate under irrigated conditions. *Indian J. Agron.* 51, 283–287 (2006).
46. Mahajan, G., Sardana, V., Brar, A.S. & Gill, M.S. 2006. Effect of seed rates, irrigation intervals and weed pressure on productivity of direct-seeded rice (*Oryza sativa*). *Indian J. Agron.* 76, 756–759 (2006).
47. Sharma, R.S., Thakur, C.L. & Agarwal, K.K. Comparison of transplanted and direct-seeded rice for productivity, profitability and physical properties of soil. *Oryza* 32, 183–187 (1995).
48. Maity, S.K. & Mukherjee, P.K. 2008. Integrated weed management in dry direct-seeded rainy season rice (*Oryza sativa*). *Indian J. Agron.* 53, 116–120 (2008).
49. Azmi, M. & Johnson, D.E. Crop establishment options for lowland irrigated rice in relation to weed infestation and grain yield. *J. Trop. Agric. Food Sci.* 37, 111–117 (2009).
50. Gangwar, K.S., Sharma, S.K., Tomer, O.K. & Pandey, D.K. Effect of crop establishment methods on hybrid rice productivity in northwest India. *Int. Rice Res. Notes* 30, 42–43 (2005).
51. Humphreys, E., Chalk, P.M., Muirhead, W.A. & White, R.J.G. Nitrogen fertilization of dry-seeded rice in south-east Australia. *Fert. Res*. **31,** 221–234 (1992).
52. Sudhir-Yadav, Gill, M.S. & Kukal, S.S. Performance of direct-seeded basmati rice in semi-arid sub-tropical India. *Soil Till. Res*. **97,** 229–238 (2007).
53. Singh, Y., Singh, G., Johnson, D. & Mortimer, M. Changing from transplanted rice to direct seeding in the rice-wheat cropping system in India. *In: Rice Is Life*: scientific perspectives for the 21st century. IRRI, Philippines.198–201 (2004).
54. De Datta, S.K. & Nantasomsaran, P. Status and prospects of direct seeded flooded rice in tropical Asia. *In: Direct seeding: status and prospects.* IRRI, Philippines. 1–16 (1991).
55. Oyediran, I.O.& Heinrichs, E.A. Arthropod populations and rice yields in direct-seeded and transplanted lowland rice in West Africa.*Int. J. Pest Manage*.**47:3,** 195–200 (2001).
56. Rahman, M.B., *et al*. Influence of N and weed management practices on rice yield under different crop establishment methods. *Eco-friendly Agric. J.* **1,** 26–31 (2008).
57. Rahman, M.B., Hossain, S.M.A., Sarkar, A.B.S., Biswas, J.C. & Islam, S.A. Growth and yield of direct wet seeded rice as affected by weed management practices. *Intl. J. BioRes*. **2,** 46–53 (2007).

58. Rahman, M.B., *et al*. Studies on the performances of wet-seeded and transplanted Aman and Boro rice. *Eco-friendly Agric. J*. **1,** 18–25 (2008).
59. Elahi, N., Saha, A., Bose, G.K., Siddiqui, M.R. & Razzaque, M.A. Direct seeding of modern rice in puddled soils: A low cost technology in rice culture. *Bangladesh J. Agric. Sci.* **22,** 1–7 (1997).
60. Elahi, N.E., Saha, A., Bose, G.K.& Razzaque, M.A. Productivity of BR varieties as transplanted and direct seeded late *Aman* crop under rainfed conditions. *Bangladesh J. Agric. Sci*. **22(2),** 291–294 (1995).
61. Saha, A., Elahi, N.E., Abedin, M.J., Quddus, M.A. & Das, T. Nitrogen doses for wet-seeded and transplanted BR14 rice variety. *Bangladesh Rice J*. **6,** 101–104 (1995).
62. Mullah, M.I.U., Bhuiys, M.S.U., Hossain, S.M.A. & Elahi, M.N. Bed planting method for establishment of direct-seeded Aman rice in rice-wheat cropping system. *Bangladesh Rice J*. **13,** 1–7 (2008).
63. Kabir, M.H., Bhuiya, M.S.U., Karim, M.M.& Elahi, N.E. Effect of crop establishment method and levels of nitrogen on the productivity of boro rice in lowland ecosystem. *Bangladesh J. Crop Sci*. **18,** 401–408 (2007).
64. Kabir, M.H., Saha, A., Mollah, I.U., Kabir, M.S. & Rahman, F. Effect of crop establishment methods and weed management practices on the productivity of boro rice in lowland ecosystem. *Intl. J. BioRes*. **5,** 42–51 (2008).
65. Islam, M.F., Sarkar, M.A.R., Islam, M.S., Parveen, S. & Hossain, M.S. Effects of crop establishment methods on root and shoot growth, lodging behavior of Aus rice. *Intl. J. BioRes*. **5,** 60–64 (2008).
66. Kabir, M.H., Bhuiya, M.S.U., Rashid, M.H., Khan, M.A.H. & Elahi, N.E. Effect of crop establishment method and weed management on the productivity of boro rice in highland ecosystem. *Intl. J. BioRes*. **4,** 52–63 (2008).
67. Kabir, M.H., Bhuiya, M.S.U., Talukder, M.A.S., Ibrahim, M. & Karim, M.M. Effect of crop establishment methods, weed control methods and nitrogen management options on the productivity of boro rice in highland ecosystem. *Bangladesh J. Prog. Sci. Technol*. **6,** 381–384 (2008)
68. Kabir, M.H., Bhuiya, M.S.U., Mollah, M.I.U., Saha, A. & Elahi, N.E. Effect of crop establishment methods, nitrogen application technique and weed management practices on the productivity of boro rice in lowland ecosystem. *Bangladesh J. Prog. Sci. Technol*. **6,** 385–388 (2008).
69. Kabir, M.H., Bhuiya, M.S.U., Karim, M.M. & Elahi, N.E. Effect of crop establishment method and levels of nitrogen on the productivity of boro rice in highland ecosystem. *Bangladesh J. Prog.Sci.Technol.***6,** 25–28 (2008).
70. Islam, M.F., Sarkar, M.A.R., Abedin, M.Z., Razzaque, M.A. & Islam, M.R. Effect of duration of seed incubation on the growth and yield of boro rice. *Bangladesh J. Crop Sci*. **18,** 19–6 (2007).
71. Islam, M.F., Sarkar, M.A.R., Islam, M.S., Parveen, S. & Mukul, H.R. Integrated weed management in direct wet-seeded aus rice using drum seeder. *Intl. J. BioRes*. **5,** 1–4 (2008).
72. Islam, M.F., Sarkar, M.A.R., Kabir, M.S., Parveen, S. & Mukul, M.H.R. Effect of rate and time of hericide application on weed control of boro rice under direct wet-seeding condition by drum seeder. *Intl. J. BioRes*. **3,** 45–49 (2007).
73. Islam, M.F., Sarkar, M.A.R., Islam, M.R., Parveen, S. & Husain, M.M. Effect of seed incubation on plant density and yield of Aman rice as affected by direct wet-seeding through drum seeder. *Intl. J. BioRes*. **2,** 40–45 (2007).

74. Islam, M.F., Sarkar, M.A.R., Abedin, M.Z., Razzaque, M.A. & Parveen, S. Effect of date of planting using drum seeder on growth and yield of boro rice. *Bangladesh J. Agric. Res*. **33,** 19–29 (2008).
75. Khan, M.A.I., *et al*. Yield performance and comparative cost analysis of wet seeded and transplanted boro rice. *Bangladesh J. Seed Sci. Technol*. **5,** 79–84 (2001).
76. Rahman, M.B., Mamun, A.A., Hossain, S.M.A., Biswas, J.C. & Mannan, M.A. Nitrogen management option for direct wet seeded rice in boro and aman sesaons. *Bangladesh Rice J*. **13,** 41–48 (2008).
77. Cabangon, R.J., Tuong, T.P. & Abdullah, N.B. Comparing water input and water productivity of transplanted and direct-seeded rice production systems. *Agric. Water Manage*. **57,** 11–31 (2002).
78. Casanova, D., Goudraiaan, J., Bouma, J. & Epema, G.F. Yield gap analysis in relation to soil properties in direct-seeded flooded rice. *Geoderma* **91,** 191–216 (1999).
79. Sharma, A.R. Direct seeding and transplanting for rice production under flood-prone lowland condition. *Field Crops Res*. **44,** 129–137 (1995).
80. Rehman, H.M., Gill, M.A., Awan, N.A. & Ladha, J.K. Evaluation and promotion of integrated crop and resource management technologies in rice-wheat systems in Pakistan. *In: J.K. Ladha, Singh, Y., Erenstein, O., Hardy, B. (Eds.). Integrated crop and resource management in the rice-wheat system of South Asia. IRRI, Philippines.* 111–132 (2009).
81. Saharawat, Y.S., *et al*. Evaluation and promotion of integrated crop and resource management in the rice-wheat system in northwest India. *In: J.K. Ladha, Singh, Y., Erenstein, O., Hardy, B. (Eds.). Integrated crop and resource management in the rice-wheat system of South Asia. IRRI, Philippines.* 133–150 (2009).
82. Singh, U.P., Singh, Y., Kumar, V. & Ladha, J.K. Evaluation and promotion of resource conserving tillage and crop establishment techniques in the rice-wheat system of eastern India. *In: J.K. Ladha, Singh, Y., Erenstein, O., Hardy, B. (Eds.). Integrated crop and resource management in the rice-wheat system of South Asia. IRRI, Philippines.* 151–176 (2009).
83. Regmi, A.P., *et al*. Improving food security through integrated crop and resource management in the rice-wheat system in Nepal. *In: J.K. Ladha, Singh, Y., Erenstein, O., Hardy, B. (Eds.). Integrated crop and resource management in the rice-wheat system of South Asia. IRRI, Philippines.* 177–196 (2009).
84. Khan, M.A.H., *et al*. Validation and delivery of improved technology in the rice-wheat ecosystem in Bangladesh. *In: J.K. Ladha, Singh, Y., Erenstein, O., Hardy, B. (Eds.). Integrated crop and resource management in the rice-wheat system of South Asia. IRRI, Philippines.* 197–220 (2009).
85. Jat, M.L., *et al*. Laser-assisted precision land leveling: a potential technology for resource conservation in irrigated intensive production systems of the Indo-Gangetic plains. *In: J.K. Ladha, Singh, Y., Erenstein, O., Hardy, B. (Eds.). Integrated crop and resource management in the rice-wheat system of South Asia. IRRI, Philippines*. 223–238 (2009).
86. Mandal, S.N., Regmi, A.P., Ladha, J.K. & Tuong, T.P. Crop establishment, tillage, and water productivity in the rice-wheat rotation in Nepal. *In: J.K. Ladha, Singh, Y., Erenstein, O., Hardy, B. (Eds.). Integrated crop and resource management in the rice-wheat system of South Asia. IRRI, Philippines.* 239–260 (2009).
87. Singh, S., *et al*. Integrated weed management: a key to success for direct-seeded rice in the Indo-Gangetic plains. *In: J.K. Ladha, Singh, Y., Erenstein, O., Hardy, B. (Eds.). Integrated crop and resource management in the rice-wheat system of South Asia. IRRI, Philippines.* 261–278 (2009).

88. Prasad, R., *et al*. Statistical tools for farmers' participatory trials for conservation agriculture. *In: J.K. Ladha, Singh, Y., Erenstein, O., Hardy, B. (Eds.). Integrated crop and resource management in the rice-wheat system of South Asia. IRRI, Philippines.* 279–296 (2009).
89. Pathak, H., *et al*. Resource-conserving technologies in the rice-wheat system of South-Asia: field evaluation and simulation analysis. *In: J.K. Ladha, Singh, Y., Erenstein, O., Hardy, B. (Eds.). Integrated crop and resource management in the rice-wheat system of South Asia. IRRI, Philippines.* 297–318 (2009).
90. Pandey, S. & Velasco, L. Economics of direct seeding in Asia: patterns of adoption and research priorities. *In: S. Pandey, Mortimer, M., Wade, L., Tuong, T.P., Lopez, K., Hardy, B. (eds.), IRRI, Philippines.* 3–14 (2002).
91. Fagi, A.M. & Kartaatmadja, S. Gogorancah rice in Indonesia: a traditional method in the modern era. *In: S.Pandey, Mortimer, M., Wade, L., Tuong, T.P., Lopez, K., Hardy, B. (eds.), IRRI, Philippines.* 43–52 (2002).
92. Tomer, V.S. The beushening system of rice crop establishment in eastern India. *In: S.Pandey, Mortimer, M., Wade, L., Tuong, T.P., Lopez, K., Hardy, B. (eds.), IRRI, Philippines.* 53–73 (2002).
93. Nguyen, D.C. & Xuan, V.T. Environmental conditions as determinants of direct seeding techniques in different ecosystems in the Mekong delta of Vietnam. *In: S. Pandey, Mortimer, M., Wade, L., Tuong, T.P., Lopez, K., Hardy, B. (eds.), IRRI, Philippines.* 75–86 (2002).
94. Ho, N.K. & Romli, Z. Impact of direct seeding on rice cultivation: lessons from Muda area of Malaysia. *In: S.Pandey, Mortimer, M., Wade, L., Tuong, T.P., Lopez, K., Hardy, B. (eds.), IRRI, Philippines.* 87–98 (2002).
95. Trebuil, G. & Thungwa, S. Farmers' direct-sowing practices in rainfed lowland rice in southern Thailand: improving a traditional system. *In: S. Pandey, Mortimer, M., Wade, L., Tuong, T.P., Lopez, K., Hardy, B. (eds.), IRRI, Philippines.* 99–114 (2002).
96. Isvilanonda, S. Development trends and farmers' benefits in the adoption of wet-seeded rice in Thailand. *In: S. Pandey, Mortimer, M., Wade, L., Tuong, T.P., Lopez, K., Hardy, B. (eds.), IRRI, Philippines.* 115–124 (2002).
97. Pandey, S., Velasco, L.E. & Suphanchalmat, N. Economics of direct seeding in northern Thailand. *In: S. Pandey, Mortimer, M., Wade, L., Tuong, T.P., Lopez, K., Hardy, B. (eds.), IRRI, Philippines.* 139–150 (2002).
98. Lee, M.H., Kim, J.K., Kim, S.S. & Park, S.T. Status of dry seeding technologies for rice in Korea. *In: S. Pandey, Mortimer, M., Wade, L., Tuong, T.P., Lopez, K., Hardy, B. (eds.), IRRI, Philippines.* 161–176 (2002).
99. Tang, S.X. Seedling broadcasting in China: an overview. *In: S. Pandey, Mortimer, M., Wade, L., Tuong, T.P., Lopez, K., Hardy, B. (eds.), IRRI, Philippines.* 177–184 (2002).
100. Mazid, M.A., Bhuiyan, S.I., Mannan, M.A. & Wade, L.J. Dry-seeded rice for enhancing productivity of rainfed drought-prone lands: lessons from Bangladesh and the Philippines. *In: S. Pandey, Mortimer, M., Wade, L., Tuong, T.P., Lopez, K., Hardy, B. (eds.), IRRI, Philippines.* 185–199 (2002).
101. Bakker, R.R., Bell, M.A. & Rickman, J.F. Mechanization issues in tillage and crop establishment for dry direct-seeded rice. *In: S. Pandey, Mortimer, M., Wade, L., Tuong, T.P., Lopez, K., Hardy, B. (eds.), IRRI, Philippines.* 219–230 (2002).
102. Du, L.V. & Tuong, T.P. Enhancing the performance of dry-seeded rice: effects of seed priming, seeding rate, and time of seeding. *In: S. Pandey, Mortimer, M., Wade, L., Tuong, T.P., Lopez, K., Hardy, B. (eds.), IRRI, Philippines.* 241–256 (2002).

103. Fukai, S. Rice cultivar requirements for direct seeding in rainfed lowlands. *In: S. Pandey, Mortimer, M., Wade, L., Tuong, T.P., Lopez, K., Hardy, B. (eds.), IRRI, Philippines.* 257–269 (2002).
104. Kyu, H., *et al.* Manipulation of seed and seedling vigor and their implications for the performance of wet-seeded rainfed lowland rice. *In: S. Pandey, Mortimer, M., Wade, L., Tuong, T.P., Lopez, K., Hardy, B. (eds.), IRRI, Philippines.* 271–280 (2002).
105. Tabbal, D.F., Bhuiyan, S.I. & Sibayan, E.B. The dry-seeding technique for saving water in irrigated rice production systems. *In: S. Pandey, Mortimer, M., Wade, L., Tuong, T.P., Lopez, K., Hardy, B. (eds.), IRRI, Philippines*. 281–297 (2002).
106. Cabangon, R.J., Tuong, T.P., Tiak, E.B. & Abdullah, N. bin. Increasing water productivity in rice cultivation: impact of the large-scale adoption of direct-seeding in the Muda irrigation system. *In: S. Pandey, Mortimer, M., Wade, L., Tuong, T.P., Lopez, K., Hardy, B. (eds.), IRRI, Philippines.* 299–313 (2002).
107. Bhattacharya, H.C., Singh, V.P. & Borkakati, K. Nutrient management of direct-seeded rice in different ecosystems of eastern India. *In: S.Pandey, Mortimer, M., Wade, L., Tuong, T.P., Lopez, K., Hardy, B. (eds.), IRRI, Philippines.* 315–330 (2002).
108. Sihavong, S.P., Sihathep, V., Inthapanya, P., Chanphengsay, M. & Fukai, S. Developing a direct-seeding technology package for rainfed lowland rice in Lao PDR. *In: S. Pandey, Mortimer, M., Wade, L., Tuong, T.P., Lopez, K., Hardy, B. (eds.), IRRI, Philippines.* 331–339 (2002).
109. Ramamoorthy, K., Balasubramanian, A. & Arokiaraj, A. Production potential and economics of direct-seeded upland rice (*Oryza sativa*) based intercropping system with grain legumes. *Indian J. Agron*. **42,** 725–726 (1997).
110. Gangwar, K.S., Gill, M.S., Tomar, O.K. & Pandey, D.K. Effect of crop establishment methods on growth, productivity and soil fertility of rice (*Oryza sativa*) based cropping systems. *Indian J. Agron*. **53,** 102–106 (2008).
111. Singh, G., *et al*. Effect of weed management practices on direct-seeded rice (*Oryza sativa*) under puddled lowlands. *Indian J. Agron*. **50,** 35–37 (2005).
112. Ojha, N.J. & Talukdar, M.C. Yield and yield attributes of direct seeded rainfed summer rice (*Oryza sativa*) as influenced by levels of potassium and sources of organic matter. *Indian J. Agric. Sci*. **70,** 774–776 (2000).
113. Gill, M.S. Productivity of direct-seeded rice (*Oryza sativa*) under varying seed rates, weed control and irrigation levels. *Indian J. Agric. Sci*. **78,** 766–770 (2008).
114. Ramakrishnan, M.S., Velayudham, K. & Ali, M. Effect of seed rate and weed management on direct-seeded lowland rice (*Oryza sativa*). *Indian J. Agron*. **37,** 808–809 (1992).
115. Kumar, S.R., Reddy,M.R., Reddy, T.M. & Reddy, B.B. Nitrogen management in direct seeded and transplanted rice. *Indian J. Agron*. **31,** 100–101 (1986).
116. Prasad, U.K., Prasad, T.N. & Kumar, A. Response of direct-seeded rice (Oryza sativa) to levels of nitrogen and irrigation in calcareous soil. *Indian J. Agron*. **37,** 686–689 (1992).
117. Thakur, R.B. Effect of sowing method and seed rate on the performance of high-yielding varieties of rice (*Oryza sativa*). *Indian J. Agron*. **38,** 547–550 (1993).
118. Ram, M., Om, H., Dhiman, S.D. & Nandal, D.P. Productivity and economics of rice (*Oryza sativa*) and wheat (*Triticum aestivum*) cropping systems as affected by establishment methods and tillage practices. *Indian J. Agron*. **51,** 77–80 (2006).
119. Singh, R.K, & Namdeo, K.N. Effect of fertility levels and herbicides on growth, yield and nutrient uptake of direct-seeded rice (*Oryza sativa*). *Indian J. Agron*. **49,** 34–36 (2004).

120. Singh, D.K., Tewari, A.N. & Tripathi, A.K. Effect of herbicides on weed dynamics and yield of direct seeded puddled rice (*Oryza sativa*) under varying water regimes. *Indian J. Agron*. **77,** 415–419 (2007).
121. Halder, J. & Patra, A.K. Performance of 8-row drum seeder in direct seeded rice (*Oryza sativa*) under puddled conditions. *Indian J. Agric. Sci*. **77,** 819–823 (2007).
122. Sharma, S.K., Pandey, D.K., Gangwar, K.S. & Chaudhry, V.P. Effect of weed management practices on productivity and profitability of direct sown unpuddled rice (*Oryza sativa*) wheat (*Triticum aestivum*) system. *Indian J. Agric. Sci*. **78,** 277–280 (2008).
123. Balasubramanian, R. & Krishnarajan, J. Weed population and biomass in direct-seeded rice (*Oryza sativa*) as influenced by irrigation. *Indian J. Agron*. **46,** 101–106 (2001).
124. Sharma, S.K., Pandey, D.K., Gangwar, K.S. & Tomer, O.K. Effect of crop establishment method on performance of rice (*Oryza sativa*) cultivars and their effect on succeeding wheat (*Triticum aestivum*). *Indian J. Agron*. **50,** 253–255 (2005).
125. Singh, Y., et al. Effect of rice (*Oryza sativa*) establishment methods, tillage practices in wheat (*Triticum aestivum*) and fertilisation on soil physical properties and rice-wheat system productivity on a silty clay Mollisol of Uttaranchal. *Indian J. Agric. Sci*. **72,** 200–205 (2002).
126. Wiangsamut, B., Mendoza, T.C. & Lafarge, T.A. Growth dynamics and yield of rice genotypes grown in transplanted and direct-seeded fields. *J. Agric. Technol*. **2,** 299–316 (2006).
127. Valarmathi, G. & Leenakumary, S. Performance analysis of high yielding rice varieties of Kerala under direct seeded and transplanted conditions. *Crop Res.***16,** 284–286 (1998).
128. Sanjay, M.T., Prabhakara Setty, T.K. & Nanjappa, H.V. Productivity, energetics and economics of different systems of crop establishment in rice. *Crop Res*. **31,** 350–353 (2006).
129. Sarma, J.S. & Dhillon, S.S. Production potential of rice (*Oryza sativa*) wheat (*Triticum aestivum*) cropping system under different methods of crop establishment. *Indian J. Agron*. **45,** 21–24 (2000).
130. Gupta, R.K., Naresh, R.K., Hobbs, P.R. & Ladha, J.K. Adopting conservation agriculture in the rice-wheat system of the Indo-Gangetic plains: new opportunities for saving water. *In: Bouman, B.A.M., Hengsdijk, H., Hardy, B., Bindraban, P.S., Tuong, T.P., Ladha, J.K. (eds.). Water-wise rice production, 8-11 April 2002, Los Baños, (Philippines: International Rice Research Institute.* 207–222 (2002).
131. Singh, A.K., Choudhury, B.U. & Bouman, B.A.M. Effect of rice establishment methods on crop performance, water use and mineral nitrogen. *In: Bouman, B.A.M., Hengsdijk, H., Hardy, B., Bindraban, P.S., Tuong, T.P., Ladha, J.K. (eds.). Water-wise rice production, 8-11 April 2002, Los Baños, (Philippines: International Rice Research Institute.* 237–246 (2002).
132. Awan, T.H., Inalyat, A., Ehsan Safdar, M., Ashraf, M.M., Yaqub, M. Economic effects of different plant establishments techniques on rice, *Oryza sativa*, production. *J. Agric. Res.* **45(1),** 73–81(2007).
133. Sanjeewanie Ginigaddara, G.A. & Ranamukhaarachchi, S.L. Effect of conventional, SRI and modified water management on growth, yield and water productivity of direct-seeded and transplanted rice in central Thailand. *Aust. J. Crop Sci*. **3(5),** 278–286 (2009).
134. Kabaki, N., *et al*. Development of a Sustainable Lowland Cropping System in Northeast Thailand. *In: Development of sustainable agricultural systems in Northeast Thailand through local resource utilisation and technology improvement. Eds., Eto, O. and Matsumoto, N. JIRCAS, Japan.* 21–127 (2002).
135. Singh, S., Sharma, S.N. & Prasad, R. The effect of seeding and tillage methods on productivity of rice-wheat cropping system. *Soil Till. Res*. **61,** 125–131 (2001).
136. Singh, T.P. Performance of no-till drill for establishment of rice and its comparison with drum seeder and conventional method. *Technical Sci*. **11,** 11–20 (2008).

137. Ahmad, M.D., Turral, H., Masih, I., Giordano, M. & Masood, Z. 2007. Water saving technologies: Myths and realities revealed in Pakistan's rice-wheat systems. Colombo, Sri Lanka: International Water Management Institute. *IWMI Research Report* **108,** 44 (2007).
138. Mazid, M.A., *et al*. Rice establishment in drought-prone areas of Bangladesh. *In: Toriyama, K., Heong, K.L., Hardy, B, editors. Rice is life: scientific perspectives for the 21st century. Proceedings of the World Rice Research Conference held in Tokyo and Tsukuba, Japan, 4-7 November 2004. Los Baños (Philippines): International Rice Research Institute, and Tsukuba (Japan): Japan International Research Center for Agricultural Sciences.* 193–195 (2005).
139. Johnson, D.E., White, J.L. & Mortimer, M. Development of sustainable weed management systems in direct-seeded, irrigated rice. *Final Technical Report. 1 April 1999 to 31 December 2002. Natural Resource Institute, University of Greenwich, Chatham, Kent, U.K.* (2003).
140. Lantican, M.A., Lampayan, R.M., Bhuiyan, S.I. & Yadav, M.K. Determination of improving productivity of dry-seeded rice in rainfed lowlands. *Exp. Agric*. **35,** 127–140 (1999).
141. Reddy, C.V., Malik, R.K. & Yadav, A. Performance of rice cultivars under different resource conservation techniques. *In: Proc. 14th Australian Agronomy Conference, 21-25 September*. 5 (2008).
142. Jehangir, W.A., Turral, H. & Masih, I. Water productivity of rice crop in irrigated areas. *In: Proc. 14th Australian Agronomy Conference, 21-25 September.* 8 (2008).
143. Wang, B., *et al*. Methane emissions from rice fields as affected by organic amendment, water regime, crop establishment, and rice culture. *Environ. Monit. Assess*. **57,** 213–228 (1999).
144. Setyanto, P., Makarim, A.K., Fagi, A.M., Wassmann R. & Buendia, L.V. Crop management affecting methane emissions from irrigated and rainfed rice in Central Java (Indonesia). *Nutr. Cycl. Agroecosys.***58,** 85–93 (2000).
145. Sharma, M.K., Sharma, R.P. & Kumar, R. Productivity and economics of rice-wheat cropping system as affected by crop establishment method and tillage practices. *J. Appl. Biol*. **17,** 56–60 (2007).
146. Qureshi, A.S., Masih, I. & Turral, H. Comparing land and water productivities of transplanted and direct dry seeded rice for Pakistani Punjab. *Zeitschrift fur Bewasserungswirtschaft* **41,** 47–60 (2006).
147. Hayashi, S., Kamoshita, A., Yamagishi, J., Kotchasati, A. & Jongdee, B. Genotypic differences in grain yield of transplanted and direct-seeded rainfed lowland rice (*Oryza sativa* L.) in northeastern Thailand. *Field Crops Res*. **102,** 9–21 (2007).
148. Rickman, J.F., Pyseth, M., Bunna, S. & Sinath, P. Direct seeding of rice in Cambodia. *ACIAR Proceedings 101, Increased lowland rice production in Mekong region, ed. by Shu Fukai and Jaya Basanyake, Cambodia.* (2001).
149. Minh, L.Q., Tuong, T.P., Mensvoort, M.E.F. van. & Bouma, J. Tillage and water management for rice land productivity in acid sulfate soils of the Mekong delta, Vietnam. *Soil Till. Res*. **42,** 1–14 (1997).
150. Mazid, M.A., Riches, C.R., Mortimer, A.M., Wade, L.J. & Johnson, D.E. 2006. Improving rice-based cropping systems in north-west Bangladesh. *Fifteenth Australian Weed Conference*. 331–334 (2006).
151. Ko, J.C., *et al*. A medium late maturing new cultivar with high grain quality, multi-disease resistance, adaptability to direct seeding and transplanting cultivation, "Hopum". *Korean J. Breed. Sci*. **40,** 533–536 (2008).
152. Baloch, M.S., Awan, I.U., Hasa, G. & Zubair, M. Studies on plant population and stand establishment techniques for increasing productivity of rice on Dera Ismail Khan. *Pakistan Rice Sci*. **14,**118–224 (2007).

153. Song, C.S., Sheng-guan, C.A.I., Xin, C. & Guo-ping, Z. Genotypic differences in growth and physiological responses to transplanting and direct seeding cultivation of rice. *Rice Sci*. **16,** 143–150 (2009).
154. Watanabe, T., *et al.* Effect of continuous rice straw compost application on rice yield and soil properties in the Mekong delta. *Soil Sci. Plant Nutr*. **55,** 754–763 (2009).
155. Supaad, M.A. & Cheong, A.W. Wet seeding in peninsular Malaysia: extent, problems, and policies. *In: K. Moody (Editor). Constraints, opportunities, and innovations for wet-seeded rice. IRRI Discussion Paper Series No. 10. International Rice Research Institute, Manila, Philippines*. 6–21 (1995).
156. Awang, Z.B. Wet seeded rice cultivation in the north-west silangor project, Malaysia. *In: K. Moody (Editor). Constraints, opportunities, and innovations for wet-seeded rice. IRRI Discussion Paper Series No. 10. International Rice Research Institute, Manila, Philippines*. 22–33 (1995).
157. Reddy, M.D., Reddy, V.N. & Rao, P.S. Wet seeded rice technology and its prospects in Andhra Pradesh. *In: K. Moody (Editor). Constraints, opportunities, and innovations for wet-seeded rice.IRRI Discussion Paper Series No. 10. International Rice Research Institute, Manila, Philippines*. 34–47 (1995).
158. Sattar, M.A. & Khan, M.A.K. An assessment of wet seeded rice cultivation technique in Bangladesh. *In: K. Moody (Editor). Constraints, opportunities, and innovations for wet-seeded rice.IRRI Discussion Paper Series No. 10. International Rice Research Institute, Manila, Philippines*. 48–58 (1995).
159. Bo, T.M. & Min, H. Wet seeded rice. *In: K. Moody (Editor). Constraints, opportunities, and innovations for wet-seeded rice. IRRI Discussion Paper Series No. 10. International Rice Research Institute, Manila, Philippines*. (1995).
160. Pandey, S. Socioeconomic research issues. *In: K. Moody (Editor). Constraints, opportunities, and innovations for wet-seeded rice. IRRI Discussion Paper Series No. 10. International Rice Research Institute, Manila, Philippines*. 73–84 (1995).
161. Ho, N.K. Management innovations and technological transfer in wet-seeded rice: A case study of the MUDA irrigation scheme, Malaysia. *In: K. Moody (Editor). Constraints, opportunities, and innovations for wet-seeded rice. IRRI Discussion Paper Series No. 10. International Rice Research Institute, Manila, Philippines*. 85–97 (1995).
162. Polvatana, A. Cultural practices in wet-seeded rice in Thailand. *In: K. Moody (Editor). Constraints, opportunities, and innovations for wet-seeded rice. IRRI Discussion Paper Series No. 10. InternationalRice Research Institute, Manila, Philippines*. 98–106 (1995).
163. Nabheerong, N. Integrated soil, crop and nutrient management practices in broadcast seeded flooded rice. *Ph.D. thesis. University of the Philippines, Los Baños, College, Laguna, Philippines*. 234p. (1990).
164. Satawathananont, S. & Boondown, S. Wet-seeded rice in problem soils. *In: K. Moody (Editor). Constraints, opportunities, and innovations for wet-seeded rice. IRRI Discussion Paper Series No. 10. International Rice Research Institute, Manila, Philippines*. 133–142 (1995).
165. Bhuiyan, S.I., Sattar, M.A. & Tabbal, D.F. Wet seeded rice: water use efficiency, productivity, and constraints to water adoption. *In: K. Moody (Editor). Constraints, opportunities, and innovations for wet-seeded rice. IRRI Discussion Paper Series No. 10. International Rice Research Institute, Manila, Philippines*. 143–155 (1995).
166. Pablico, P.P., Yamauchi, M., Tuong, T.P., Cabangon, R.J. & Moody, K. Crop establishment and weed competitiveness of direct seeded anaerobic rice cultivar as influenced by seeding and water management systems. *In: K. Moody (Editor). Constraints, opportunities, and innovations*

for wet-seeded rice. IRRI Discussion Paper Series No. 10. International Rice Research Institute, Manila, Philippines. 156–170 (1995).

167. Yamauchi, M., *et al*. Rice anaerobic direct seeding in the tropics. *In: K. Moody (Editor). Constraints, opportunities, and innovations for wet-seeded rice. IRRI Discussion Paper Series No. 10. International Rice Research Institute, Manila, Philippines.* 171–185 (1995).
168. Chuong, P.V. & Yamauchi, M. Anaerobic direct seeding of rice in northern Vietnam. *In: K. Moody (Editor). Constraints, opportunities, and innovations for wet-seeded rice. IRRI Discussion Paper Series No. 10. International Rice Research Institute, Manila, Philippines.* 186–198 (1995).
169. Bridgit, T.K. & Mathew, J. Differential response of rice to coated and enlarged urea forms under wet seeded and transplanted systems. *In: K. Moody (Editor). Constraints, opportunities, and innovations for wet-seeded rice. IRRI Discussion Paper Series No. 10. International Rice Research Institute, Manila, Philippines*. 219–230 (1995).
170. Kundu, D.K., Rao, K.V. & Pillai, K.G. Use of green manures as nitrogen sources for wet seeded rice. *In: K. Moody (Editor). Constraints, opportunities, and innovations for wet-seeded rice. IRRI Discussion Paper Series No. 10. International Rice Research Institute, Manila, Philippines.* 231–245 (1995).
171. Garcia, F.V., Peng, S. & Cassman, K.G. Yield potential of transplanted and wet seeded rice in high yield environments in the Philippines. *In: K. Moody (Editor). Constraints, opportunities, and innovations for wet-seeded rice. IRRI Discussion Paper Series No. 10. International Rice Research Institute, Manila, Philippines.* 246–256 (1995).
172. Hassan, S.M., Rao, A.N., Bastawasi, A.O. & Aidy, I.R. Weed management in wet seeded rice in Egypt. *In: K. Moody (Editor). Constraints, opportunities, and innovations for wet-seeded rice. IRRI Discussion Paper Series No. 10. International Rice Research Institute, Manila, Philippines.* 257–269 (1995).
173. Vongsaroj, P. Weed control in wet seeded rice in Thailand. *In: K. Moody (Editor). Constraints, opportunities, and innovations for wet-seeded rice. IRRI Discussion Paper Series No. 10. International Rice Research Institute, Manila, Philippines.* 270–286 (1995).
174. Gogoi, A.K., Upadhyay, U.C. & Roy, A.K. Status of weed management in wet seeded rice in Assam, India. *In: K. Moody (Editor). Constraints, opportunities, and innovations for wet-seeded rice. IRRI Discussion Paper Series No. 10. International Rice Research Institute, Manila, Philippines.* 298–310 (1995).
175. Purushothaman, S. & Ilangovan, R. Studies on integrated weed management and cultivar screening for herbicide tolerance in wet seeded rice. *In: K. Moody (Editor). Constraints, opportunities, and innovations for wet-seeded rice. IRRI Discussion Paper Series No. 10. International Rice Research Institute, Manila, Philippines.* 311–323 (1995).
176. Bazaya, B.R.,Sen, A.,& Srivastava, V.K. Planting methods and nitrogen effects on crop yield and soil quality under direct seeded rice in the Indo-Gangetic plains of eastern India. *Soil Till. Res*. **105,** 27–32 (2009).
177. Tripathi, J., Bhatta, M.R., Justice, S. & Shakya, N.K. Direct-seeding: an emerging resource conserving technology for ricecultivation in the rice wheat system. *In: Proceedings of Rice Reseach (sent by Dr.Virender in PDF format).* 273–281 (2004).
178. Sah, G. & Sah, R.P. Evaluation of low cost direct seeded rice technologies in farmers' fields. *In: Proceedings of Rice Research.* 390–396 (2004).
179. Ramesh, S. & Chandrasekharan, B. Crop establishment methods and nitrogen management strategies on realising yield potential of rice hybrid ADTRH 1. *Asian J. Plant Sci*. **6,** 239–251 (2007).

180. Adhikari, N.P., Gautam,A.K.,Pradhan, G., Bhattarai, E.M. & Das, R.B. Effect of tillage and crop establishment on grain yield under rice-wheat system. *Proceedings of Rice Research (PDF file sent by Mr.Virender).* (2004).
181. Khiem, T. Grass control in the directly sowed rice fields by watering. *Nong Nghiep Cung Nghiep Thug Pham* **6,** 221–223 (1992).
182. Du, P.V., Cuong, N.L. & Chau, L.M. Effect of herbicides on*Echinochloa* spp. in lowland rice of Mekong delta. *In: Selected papers. Mekong Delta Rice Research Institute, Ministry of Agriculture and Food Industry, Oman, Hau Giang, Vietnam.* 53–59 (1990).
183. Baki, B.B. & Azmi, M. Integrated management of paddy and aquatic weeds in Malaysia: Current status and prospects for improvement. *In: Proc. FFTC International Symposium on biological and integrated management of paddy and aquatic weeds in Asia. 19-24 October, Tsukuba, Japan.* 105–160 (1992).
184. Supaad, M.A., Othman, O. & Cheong, A.W. Scenario and prospects of direct seeding in Malaysia. *In: Proc. National rice conference of direct seeding practices and productivity (Ali Abdul Hamid, Editor), MARDI, Seberang Perai, Malaysia*. 29–49 (1990).
185. Sipaseuth, P.I., *et al*. Agronomic practices for improving yields of rainfed lowland rice in Laos. *In: ACIAR Proceedings 101. Increasing lowland rice production in the Mekong region (ed. by Shu Fukai and Jaya Basnayake).* 31–40 (2001).
186. Abdul Shukor Juraimi, Mohamad Najib, M.Y., Begum, M.,& Anuar, A.R. Critical period of weed competition in direct seeded rice under saturated and flooded conditions. *Pertanika J. Trop. Agric. Sci.* **32,** 305–316 (2009).
187. S. Kartaatmadja., *et al*. Optimizing use of natural resources and increasing rice productivity. *In: 13th International Soil Conservation Organisation Conference - Conserving Soil and Water for Society: Sharing Solutions, Brisbane*. **758**, 4p. (2004).
188. Jeyanny, V., Omar, S.R.S., Azmi, M. & Juraimi, A.S. Enhancing rice establishment in anaerobic direct seeding through control of weedy rice. *Malaysian J. Soil Sci*. **12,** 77–86 (2008).
189. Tran Quang Tuyen & Truong Thi Ngoc Chi. Farmers' nutrient management in intensive irrigated and direct seeded rice production system in Mekong delta, Vietnam. *Omon Rice***13,** 90–96 (2005).
190. Min Huang, *et al*. Effect of tillage on soil and crop properties of wet-seeded flooded rice. *Field Crops Res*. **129,** 28–38 (2012).
191. Min Huang, *et al*. No tillage and direct seeding for super hybrid rice production in rice-oilseed rape cropping system.*Eur. J. Agron.***34,** 278–286 (2011).
192. Mahajana, G., Chauhanb, B.S., Timsinab, J., Singha, P.P. & Kuldeep Singha. Crop performance and water- and nitrogen-use efficiencies in dry-seeded rice in response to irrigation and fertilizer amounts in northwest India. *Field Crops Res*. **134,** 59–70 (2012).
193. Sudhir-Yadav, Gill, G., Humphreys, E., Kukal, S.S. & Walia, U.S. Effect of water management on dry seeded and puddled transplanted rice. Part 1: Crop performance. *Field Crops Res*. 112–122 (2011).
194. Albertoa, M.C.R.,*et al*. Carbon uptake and water productivity for dry-seeded rice and hybrid maize grown with overhead sprinkler irrigation. *Field Crops Res*. **146,** 51–65 (2013).
195. Chauhan, B.S. & Opena, J. Implications of plant geometry and weed control options in designing a low-seeding seed-drill for dry-seeded rice systems. *Field Crops Res*. **144,** 225–231 (2013).
196. Dusserrea, J.,Choparta, J.-L.,Douzeta, J.-M., Rakotoarisoab, J.,& Scopela, E. Upland rice production under conservation agriculture cropping systems in cold conditions of tropical highlands. *Field Crops Res*. **138,** 33–41(2012).

197. Devkota, K.P., *et al*. Growth and yield of rice (*Oryza sativa* L.) under resource conservation technologies in the irrigated drylands of Central Asia. *Field Crops Res*. **149,** 115–126 (2013).
198. Clerget, B.,*et al*. Modifications in development and growth of a dual-adapted tropical rice variety grown as either a flooded or an aerobic crop. *Field Crops Res*. **155,** 134–143 (2014).
199. Jing Yan, *et al*. Yield formation and tillering dynamics of direct-seeded rice in flooded and non-flooded soils in the Huai River Basin of China. *Field Crops Res*. **116,** 252–259 (2010).
200. Ranjan Laik, et al. Integration of conservation agriculture with best management practices for improving system performance of the rice-wheat rotation in the Eastern Indo-Gangetic Plains of India.*Agric. Ecosys. Environ.***195,** 68–82 (2014).
201. Gathala, M.K., *et al*. Physical and chemical properties of a sandy loam soil under irrigated rice-wheat sequence in the Indo-Gangetic Plains of South Asia. *J. Ecosys. Ecograph*. **S7**, 002. doi: 10.4172/2157-7625.S7-002 (2017).
202. Okami, M., Kato, Y.,&Yamagishi, J. Role of early vigor in adaptation of rice to water-saving aerobic culture: Effects of nitrogen utilization and leaf growth. *Field Crops Res*. **124,** 124–131(2011).
203. Meisner, C.A., *et al.*Nitrogen and irrigation management for direct-seeded rice on light soils in a rice-wheat cropping system. *In: Proceedings 17th World Congress of Soil Sciences (WCSS)*. 14–21(2002).
204. McDonald, A.J., Riha, S.J., Duxbury, J.M. & Lauren, J.G. Wheat responses to novel rice cultural practices and soil moisture conditions in the rice–wheat rotation of Nepal. *Field Crops Res*. **98,** 116–126 (2006).
205. Parihar, S.S. Effect of crop-establishment method, tillage, irrigation and nitrogen on production potential of rice-wheat cropping system. *Indian J. Agron*. **49,** 1–5 (2004).
206. Dhiman, S.D., Sharma, H.C., Nandal, D.P., Om, H. & Singh, D. Effect of irrigation, methods of crop establishment and fertilizer management on soil properties and productivity in rice (*Oryza sativa*)-wheat (*Triticum aestivum*) sequence. *Indian J. Agron*. **43,** 208–212 (1998).
207. Malik, R.K., Yadav, A. & Singh, S. Resource conservation technologies in rice–wheat cropping system of Indo-Gangetic Plain. *In: "Conservation Agriculture: Status and Prospects" in Abrol, I.P., R.K. Gupta, and R.K. Malik (Editors). Conservation Agriculture – Status and Prospects. Centre for Advancement of Sustainable Agriculture, New Delhi,* 13–22 (2005).
208. Gangwar, K.S., Chaudhary, V.P., Gangwar, B. & Pandey, D.K. Effect of crop establishment and tillage practices in rice (*Oryza sativa*)-based cropping systems. *Indian J. Agric. Sci*. **79,** 334–339 (2009).
209. Sharma, P., Tripathi, R.P., Singh, S. & Kumar, R. Effects of tillage on soil physical properties and crop performance under rice-wheat system. *J. Indian Soc. Soil Sci*. **52,** 12–16 (2004).
210. Tripathi, R.P., Sharma, P. & Singh, S. Tillage index: An approach to optimize tillage in rice-wheat system. *Soil Till. Res*. **80,** 125–137 (2005).
211. Corton, T.M., *et al*. Methane emission from irrigated and intensively managed rice fields in Central Luzon (Philippines). *Nutr. Cycl. Agroecosyst*. **58,** 37–53 (2000).
212. Singh, S.K., *et al*. Influence of crop establishment methods on methane emission from rice fields. *Curr. Sci.***97,** 84–89 (2009).
213. Saharawat, Y.S., *et al*. Evaluation of alternative tillage and crop establishment methods in a rice–wheat rotation in North Western IGP. *Field Crops Res*. **116,** 260–267 (2010).
214. Mishra, J.S. & Singh, V.P. Tillage and weed control effects on productivity of a dry seeded rice–wheat system on a Vertisol in Central India. *Soil Till. Res.***123,** 11–20 (2012).
215. Chauhan, B.S. & Opena, J. Effect of tillage systems and herbicides on weed emergence, weed growth, and grain yield in dry-seeded rice systems. *Field Crops Res.***137,** 56–69 (2012).

216. Chauhan, B.S. & Opena, J. Weed management and grain yield of rice sown at low seeding rates in mechanized dry-seeded systems. *Field Crops Res*. **141,** 9–15 (2013).

217. Singh, S., Ladha, J.K., Gupta, R.K., Lav Bhushan & Rao, A.N. Weed management in aerobic rice systems under varying establishment methods. *Crop Prot*. **27,** 660–671 (2008).

218. Singh, S., *et al*. Weed management in dry-seeded rice (*Oryza sativa* l.). *Crop Prot*. **26,** 518–524 (2007).

219. Singh, S., *et al*. Evaluation of mulching, intercropping with Sesbania and herbicide use for weed management in dry-seeded rice (*Oryza sativa*L.) cultivated in the furrow-irrigated raised-bed planting system. *Crop Prot.***25,** 487–495 (2006).

220. Singh, Y., *et al*. The implications of land preparation, crop establishment method and weed management on rice yield variation in the rice-wheat system in the Indo-Gangetic plains. *Field Crops Res*. (2011).

221. Sarangi, Sukanta K., *et al*. Crop establishment and nutrient management for dry season (boro) rice in coastal areas. *Agron. J.* **106,** 2013–2023 (2014).

222. Chauhan, B.S. & Johnson, D.E. Row spacing and weed control timing affect yield of aerobic rice. *Field Crops Res*. **121,** 226–231 (2011).

223. Farooq, M., *et al*. Rice direct seeding: Experiences, challenges and opportunities. *Soil Till. Res*. **111,** 87–98 (2011).

224. Gathala, M.K., *et al*. Tillage and crop establishment affect sustainability of South Asian rice–wheat system. *Agron. J*. **103,** 961–971 (2011).

225. Gathala, M.K.,*et al*. Optimizing intensive cereal-based cropping systems addressing current and future drivers of agricultural change in the north western Indo-Gangetic Plains of India. *Agric. Ecosyst. Environ*.**187,** 33–46 (2014).

226. Griggs, B.R., Norman, R.J., Wilson Jr., C.E. & Slaton, N.A. Ammonia volatilization and nitrogen uptake for conventional and conservation tilled dry-seeded, delayed-flood rice. *Soil Sci. Soc. Am. J*.**71,** 745–751 (2007).

227. Ko, J.Y. & Kang, H.W. The effects of cultural practices on methane emission from rice fields. *Nutr. Cycl. Agroecosyst*. **58,** 311–314 (2000).

228. Mahajan, G., Chauhan, B.S. & Johnson, D.E. Weed management in aerobic rice in north western Indo-Gangetic Plains. *J. Crop Improve*. **23,** 366–382 (2009).

229. Mahajan, G., Gill, M.S. & Singh, K. Optimizing seed rate to suppress weeds and to increase yield in aerobic direct seeded rice in north western Indo-Gangetic Plains. *J. New Seeds* **11,** 225–238 (2010).

230. Mahajan, G. & Timsina, J. Effect of nitrogen rates and weed control methods on weeds abundance and yield of direct-seeded rice. *Arch. Agron. Soil Sci*. **57:3,** 239–250 (2011).

231. Naresh, R.K., *et al*. Experiences with rice grown on permanent raised beds: Effect of crop establishment techniques on water use, productivity, profitability and soil physical properties. *Rice Sci*. **21(3),** 170−180 (2014).

232. Norman, R.J., *et al*. Nitrogen fertilizer sources and timing before flooding dry-seeded, delayed-flood rice. *Soil Sci. Soc. Am. J*.**73,** 2184–2190 (2009).

233. Sharma, A.R. & Ghosh, A. Effect of green manuring with *Sesbania aculeata* and nitrogen fertilization on the performance of direct-seeded flood-prone lowland rice. *Nutr. Cycl. Agroecosyst*. **57,** 141–153 (2000).

234. Jacob, G., Menon, M.V. & Abraham, C.T. Comparative efficacy of new herbicides in direct seeded rice. *J. Trop. Agric*. **52 (2),** 174–177 (2014).

235. Bhurer, K.P., Yadav, D.N., Ladha, J.K., Thapa, R.B. & Pandey, K. Effect of integrated weed management practices on performance of dry direct seeded rice (*Oryza sativa* L.). *Agron. J. Nepal***3,** 53–63 (2013).

236. Sanusan, S., *et al*. Suppressing weeds in direct-seeded lowland rainfed rice: Effect of cutting dates and timing of fertilizer application. *Crop Prot*. **29,** 927–935 (2010).
237. Zhang, J.-S., *et al*. Emissions of N_2O and NH_3, and nitrogen leaching from direct seeded rice under different tillage practices in central China. *Agric. Ecosyst. Environ*. **140,** 164–173 (2011).
238. Gathala, M.K., *et al.* Conservation agriculture based tillage and crop establishment options can maintain farmers' yields and increase profits in South Asia's rice–maize systems: Evidence from Bangladesh. *Field Crops Res*. **172,** 85–98 (2015).
239. Jat, R.K., *et al*. Seven years of conservation agriculture in a rice–wheat rotation of Eastern Gangetic Plains of South Asia: Yield trends and economic profitability. *Field Crops Res*. **164,** 199–210 (2014).
240. Liu, H., *et al*. Dry direct-seeded rice as an alternative to transplanted-flooded rice in Central China. *Agron. Sustain. Dev*. **35,** 285–294 (2014).
241. Singh, M., Bhullar, M.S. & Chauhan, B.S. Influence of tillage, cover cropping, and herbicides on weeds and productivity of dry direct-seeded rice. *Soil Till. Res*. **147,** 39–49 (2015).
242. Sidhu, A.S., Kooner, R. & Verma, A. On-farm assessment of direct-seeded rice production system under central Punjab conditions. *J. Crop Weed***10(1),** 56–60 (2014).
243. Mann, R.A., Ahmad, S., Hassan, G.& Baloch, M.S. Weed management in direct seeded rice crop. *Pak.J. Weed Sci. Res*. **13(3-4),** 219–226 (2007).
244. Farooq, M., Basra, S.M.A., Tabassum, R. & Afzal, I. Enhancing the performance of direct seeded fine rice by seed priming. *Plant Prod. Sci*. **9,** 446–456 (2006)
245. Kumar, V. & Ladha, J.K. Direct seeded rice: Recent developments& future research needs. *Adv. Agron.* **111,** 297–413 (2011).
246. Singh, Y., Singh, G., Johnson, D. & Mortimer, M. Changing from transplanted rice to direct seeding in the rice–wheat cropping system in India. *In: Rice Is Life: Scientific Perspectives for the 21st Century, Tsukuba, Japan: Proceedings of the World Rice Research Conference, 4–7 November*. 198–201 (2004).
247. Weerakoon, W.M.W., *et al*. Direct-seeded rice culture in Sri Lanka. *Field Crops Res*. **121,** 53–63 (2011).
248. Ali, R.I., Saleem, M.U., Iqbal, N.& Akhter, M. Effective weed management in dry direct seeded rice for sustainable productivity. *Pak. J. Weed Sci. Res*. **20(4),** 519–529 (2014).
249. Hussain, S., Ramzan, M., Akhter, M. & Asalam, M. Weed management in direct seeded rice. *J. Anim. Plant Sci*. **18(2-3),** 86–88 (2008).
250. Sekiya, H.,*et al*. The rice direct-seeding system using multiple seed pellets in northern Tohoku. In: Rice is life: Scientific perspectives for the 21st century. *Proceedings of the World Rice Research Conference held in Tokyo and Tsukuba. Japan. 4-7 November 2004. Los Baños (Philippines): Toriyama, K.,Heong, K.L., Hardy, B. (editors). 2005. International Rice Research Institute, and Tsukuba (Japan): Japan International Research Center for Agricultural Sciences. CD-ROM*. **590,** 241–244 (2005).
251. Mana, R.G., *et al*. Village-level modeling of environment-friendly and appropriate technologies and practices for direct seeding. In: Rice is life: Scientific perspectives for the 21st century. *Proceedings of the World Rice Research Conference held in Tokyo and Tsukuba. Japan. 4-7 November 2004. Los Baños (Philippines): Toriyama, K.,Heong, K.L., Hardy, B. (editors). 2005. International Rice Research Institute, and Tsukuba (Japan): Japan International Research Center for Agricultural Sciences. CD-ROM.***590,** 217–221(2005).
252. Goang Hui Xie, Jun Yu, Ling Yan, Huaoi Wang & Xiurong Mu. Direct seeding of aerobic rice in China. In: Rice is life: Scientific perspectives for the 21st century. *Proceedings of the World Rice Research Conference held in Tokyo and Tsukuba. Japan. 4-7 November 2004. Los Baños (Philippines): Toriyama, K., Heong, K.L., Hardy, B. (editors). 2005. International Rice*

Research Institute, and Tsukuba (Japan): Japan International Research Center for Agricultural Sciences. CD-ROM. **590,** 186–188 (2005).
253. Abdullah, Md.& Mamun, Al. Modelling rice-weed competition in direct-seeded rice cultivation. *Agric.Res*.**3(4)**, 346–352 (2014).
254. Ahmed, S.,&Chauhan, B.S. Performance of different herbicides in dry-seeded rice in Bangladesh. *Sci. World J*. **729418,** 14p. (2014).
255. Samuel, P.L., Nida, Q.A., Alma, C.A., Reynaldo, C.C. &Sebastian, L.S. Modified dry-seeding with zero tillage & straw mulching: A new technology package for rainfed rice. *Philipp. J. Crop Sci*. **26(2),** 5–13 (2001).
256. Mahajan, G. & Chauhan, B.S. Weed control in dry direct-seeded rice using tank mixtures of herbicides in South Asia. *Crop Prot*. **72,** 90–96 (2015).
257. Khaliq, A., *et al*. Supplementing herbicides with manual weeding improves weed control efficiency, growth and yield of direct seeded rice. *Int. J. Agric. Biol*. **15,** 191–199 (2013).
258. Brar, H.S.,& Bhullar, M.S. Nutrient uptake by direct seeded rice and associated weed control. *Indian J. Agric. Res*. **47(4),** 353–358 (2013).
259. Ihsan, M.Z.,*et al*. Influence of herbicides applied alone or supplemented with manual weeding on weed growth, rice yield and grain quality in direct seeded rice (*Oryza sativa* L.). *Philipp. Agric. Sci*. **97(4),** 395–402 (2014).
260. Ganie, Z.A., Singh, S.& Singh, S. Effect of seed rate and weed control methods on yield of direct seeded rice (*Oryza sativa*). *Indian J. Agron*. **58(1),** 125–126 (2013).
261. Khaliq, A., Matloob, A., Ahmad, N., Rasul, F. & Awan, I.U. Post emergence chemical weed control in direct seeded fine rice. *J. Anim. Plant Sci*. **22(4),** 1101–1106 (2012).
262. Akbar, N.E., Jabran, K.& Ali, M.A. Weed management improves yield and quality of direct seeded rice. *Aust. J. Crop Sci*. **5(6),** 688–694 (2011).
263. Awan, T.H., Safdar, M.E., Manzoor, Z. & Ashraf, M.M. Screening of herbicides as post-emergence application for effective weed control without affecting growth and yield of direct seeded rice plant. *J. Anim. PlantSci*. **16(1-2),** 60–65 (2006).
264. Thomas, R.R.& Posner, J.L. On-farm evaluation of weed control technologies in direct-seeded rice in The Gambia. *Anim. Power Weed Control* 255–261(2000).
265. Md. Abdullah Al Mamun, Rakiba Shultana, Md. Masud Rana, Abdul Jalil Mridha. Economic threshold density of multi species weed for direct seeded rice. Asian J. Agric. Rural Dev. **3(8),** 523–531 (2013).
266. Jabran, K., *et al*. Efficient weeds control with penoxsulam application ensures higher productivity and economic returns of direct seeded rice. *Int. J. Agric. Biol*. **14,** 901–907 (2012).
267. Casimero, M.C. *et.al*. Participatory adaptation of an Integrated Weed Management strategy for wet direct-seeded rice in the Philippines. *Fifteenth Australian Weeds Conference*. (2006).
268. Chauhan, B.S.&Bajwa, A.A. Management of *Rottboellia cochinchinensis* and other weeds through sequential application of herbicides in dry direct-seeded rice in the Philippines. *Crop Prot*. **78,** 131−136 (2015).
269. Rehman, H.U., Basra, S.M.A. & Farooq, M. Field appraisal of seed priming to improve the growth, yield, and quality of direct seeded rice. *Turkish J. Agric. Forestry* **35(4),** 357–365 (2011).
270. Mahajan, G; Chauhan, B.S. & Gill, M.S. Dry-seeded rice culture in Punjab State of India: Lessons learned from farmers. *Field Crops Res*. **144,** 89–99 (2013).
271. Mann, R.A., Hussain, S. & Saleem, M. Impact of dry seeding with alternate wetting and drying on rice productivity and profitability in Punjab-Pakistan. *Pak. J. Weed Sci. Res*.

272. Naresh, R.K., *et al*. Tillage crop establishment strategies and soil fertility management: Resource use efficiencies and soil carbon sequestration in a rice-wheat cropping system. *Ecol. Environ. Conserv*. **21,** 121–128 (2015).
273. Xinping Chen, *et al*. Producing more grain with lower environmental costs.doi:10.1038/nature13609 (2014).
274. Maung Maung Yi, *et al*. Disseminating integrated natural resource management for lowland rice in Myanmar. *Research to impact: Case studies for natural resource management for irrigated rice in Asia.* Edited by F.G. Palis, G.R. Singleton, M.C. Casimero, and B. Hardy. 67–81 (2010).
275. Mazid, M.A. & Johnson, D.E. Tackling hunger through early rice harvests in northwest Bangladesh: Making a difference with direct seeding and varietal choice. *Research to impact: Case studies for natural resource management for irrigated rice in Asia*. Edited by F.G. Palis, G.R. Singleton, M.C. Casimero, and B. Hardy. 83–100 (2010).
276. Singh, V.P., Pratibha Singh, Singh, Y., Malabayabas, A.J. & Johnson, D.E. Developing direct-seeding options for rice farmers in the Indo-Gangetic Plains. *Research to impact: Case studies for natural resource management for irrigated rice in Asia* Edited by F.G. Palis, G.R. Singleton, M.C. Casimero, and B. Hardy. 101–113 (2010).
277. Islam, A.K.M.S., Haque, M.E., Hossain, M.M., Saleque, M.A. & Bell, R.W. Evaluation of the versatile multi-crop planter for establishing sprouted direct-seeded rice. *5th World Congress of Conservation Agriculture incorporating 3rd Farming Systems Design Conference*, *Brisbane, Australia*. (2011).
278. Raman, A., Ladha, J.K., Kumar, V., Sharma, S.& Piepho, H.P. Stability analysis of farmer participatory trials for conservation agriculture using mixed models. *Field Crops Res*. **121,** 450–459 (2011).
279. Halder, J., Sahoo, K.C., Karmaker S.K. & Nayak, R.N. Productivity and sustainability of different crop establishment methods for cultivation of rabi rice in western Orissa *Ann. Agric. Res*. **30(3&4),** 82–86 (2009).
280. Ali, A.M., Thind, H.S., Virinderpal Singh & Bijay Singh. A framework for refining nitrogen management in dry direct seeded rice using Green Seeker. *Computers and Electronics in Agric*. **110,** 114–120 (2015).
281. Kukal, S.S., Bhatt, R., Gupta, N.& Singh, M.C. Effect of crop establishment methods on performance of rice (*Oryza sativa* L.) and irrigation water productivity in sandy-loam soil. *J. Res. Punjab Agric. Univ*. **51(3&4),** 326–328 (2014).
282. Shyam Prakash, M., G., Sharma, N. & Sardana, V. Enhancing grain yield and nitrogen-use efficiency in rice through foliarly applied gibberellic acid in dry-direct-seeded rice. *J. Crop Improve*.**29(1),** 65–81 (2015).
283. Rahman, M.M.,& Masood, M.M. Sustaining productivity in boro (winter) season using minimal water through dry direct seeding of rice. *J. Crop Weed* **10(2),** 24–30 (2014).
284. Rehman, H.U., Basra, S.M.A. & Wahid, A. Optimizing nitrogen-split application time to improve dry matter accumulation and yield in dry direct seeded rice. *Int. J. Agric. Biol*., **15,** 41–47 (2013).
285. Sudhir-Yadav, *et al*. Establishment method effects on crop performance and water productivity of irrigated rice in the tropics. *Field Crops Res.***166**, 112–127 (2014).
286. Ekta J., *et al*. Management of direct seeded rice for enhanced resource-use efficiency. *Plant Knowl. J.* **2(3),** 119–134 (2013).
287. Gill, J.S.& Walia, S.S. Influence of FYM, brown manuring and nitrogen levels on direct seeded and transplanted rice (*Oryza sativa* L.): A review. *Res. J. Agric. Environ. Manage.* **3(9),** 417–426 (2014).

288. Pandey, M., Joshi, R.& Kumar, N. Crop management strategies and practices for improving water productivity in dry-seeded rice. *Int. J. Food Agric. Vet. Sci.* **5 (1),** 106–111(2015).
289. Parameswari, Y.S. & Srinivas, A. Influence of weed management practices on nutrient uptake and productivity of rice under different methods of crop establishment. *J. Rice Res.* **7(1&2),** 77–86 (2014).
290. Sompaew, V. & De Datta, S.K. Stand establishment techniques and management practices for direct-seeded rice. *Philipp. J. Crop Sci.* **5(3),** 112–116 (1980).
291. Kumara, A., Nayak, A.K., Mohanty, S.& Das, B.S. Greenhouse gas emission from direct seeded paddy fields under different soil water potentials in eastern India. *Agric. Ecosyst. Environ.* **228,** 111–123 (2016).
292. Pathak, H., Sankhyan, S., Dubey, D.S., Bhatia, A. & Jain, N. Dry direct-seeding of rice for mitigating greenhouse gas emission: Field experimentation and simulation. *Paddy Water Environ*. https://www.researchgate.net/publication/256378157. (2012).
293. Shuwei Liu, Yaojun Zhang, Feng Lin, Ling Zhang & Jianwen Zou. Methane and nitrous oxide emissions from direct-seeded and seedling-transplanted rice paddies in southeast China. *Plant Soil*. https://www.researchgate.net/publication/259634974. (2014).
294. Pathak, H. *et al*. Direct-seeded rice: Potential, performance and problems – a review. *Curr. Adv. Agric. Sci*. **3(2),** 77–88 (2011).
295. Gupta, D.K.,*et al*. Mitigation of greenhouse gas emission from rice–wheat system of the Indo-Gangetic plains: Through tillage, irrigation and fertilizer management. *Agric. Ecosyst. Environ*. **230,** 1–9 (2016).
296. Chandrasekhararao, C., Jitendranath, S. & Murthy, T.G.K. Resource optimisation in rice through direct seeding by drum seeder. *Int. J. Agric. Food Sci. Technol*. **4(3),** 239–246 (2013).
297. Mohanty, S.R. *et al*. Effect of the herbicide butachlor on methane emission and ebullition flux from a direct-seeded flooded rice field. *Biol. Fertil. Soils* **33,** 175–180 (2001).
298. Kreye, C.*et al*. Fluxes of methane and nitrous oxide in water-saving rice production in north China. *Nutr. Cycl. Agroecosyst.***77,** 293–304 (2007).
299. Ye Tao, Qian Chen, Shaobing Peng, Weiqin Wang & Lixiao Nie. Lower global warming potential and higher yield of wet direct-seeded rice in Central China. *Agron. Sustain. Dev.* **36,** 24 (2016).
300. Vijay Singh, Jat, M.L., Ganie, Z.A., Chauhan, B.S.& Gupta, R.K. Herbicide options for effective weed management in dry direct seeded rice under scented rice-wheat rotation of western Indo-Gangetic Plains. *Crop Prot.* **81,** 158–175 (2015).
301. Tripathi, R.S., Raju, R. & Thimmappa, K. Economics of direct seeded and transplanted methods of rice production in Haryana. *Oryza* **51(1),** 70–73 (2014).
302. Kumar, A., Kumar, S., Dahiya, K., Kumar, S.& Kumar, M.Productivity and economics of direct seeded rice (*Oryza sativa* L.). *J. Appl. Nat. Sci*. **7(1)**, 410–416 (2015).
303. Regional Development and Dissemination of Climate-Resilient Rice Varieties for Water-Short Areas of South Asia and Southeast Asia (Financed by the Climate Change Fund and the Government of Finland), Technical Assistance Consultant's Report 2016 Prepared by *International Rice Research Institute and partner organizations in Bangladesh, India, Nepal, Pakistan, and Philippines, Los Baños, Laguna; for the Asian Development Bank.* (2016).
304. Long-Term Experiments, IRRI, Philippines.
306 to 323. Ramesha *et al.* 2014, Hyderabad, India (personal communication).
